# Antibiotics in the global river system arising from human consumption

**DOI:** 10.1093/pnasnexus/pgaf096

**Published:** 2025-04-22

**Authors:** Heloisa Ehalt Macedo, Bernhard Lehner, Jim A Nicell, Usman Khan, Eili Y Klein

**Affiliations:** Department of Geography, McGill University, Montreal, QC, Canada H3A 0B9; Department of Geography, McGill University, Montreal, QC, Canada H3A 0B9; Department of Civil Engineering, McGill University, Montreal, QC, Canada H3A 0C3; Department of Civil Engineering, McGill University, Montreal, QC, Canada H3A 0C3; One Health Trust, Washington, DC 20015, USA; Department of Emergency Medicine, Johns Hopkins School of Medicine, Baltimore, MD 21287, USA

**Keywords:** antibiotics, water quality, model, global, rivers

## Abstract

The presence of antibiotics in surface waters poses risks to aquatic ecosystems and human health due to their toxicity and influence on antimicrobial resistance. After human consumption and partial metabolism, antibiotic residues are excreted and undergo complex accumulation and decay processes along their pathway from wastewater to natural river systems. Here, we use a global contaminant fate model to estimate that of the annual human consumption of the 40 most used antibiotics (30,300 tonnes), 9,500 tonnes (31%) are released into the river system and 3,250 tonnes (11%) reach the world's oceans or inland sinks. Even when only domestic sources are considered (i.e. not including veterinary or industrial sources), we estimate that 6 million km of rivers worldwide are subject to total antibiotic concentrations in excess of thresholds that are protective of ecosystems and resistance promotion during low streamflow conditions, with the dominant contributors being amoxicillin, ceftriaxone, and cefixime. Therefore, it is of concern that human consumption alone represents a significant risk for rivers across all continents, with the largest extents found in Southeast Asia. Global antibiotic consumption has grown rapidly over the last 15 years and continues to increase, particularly in low- and middle-income countries, requiring new strategies to safeguard water quality and protect human and ecosystem health.

**Significance Statement**

Antibiotic contamination in global rivers poses significant threats to aquatic ecosystems and human health. Our model predicts that 8,500 tonnes of antibiotics enter river systems annually from domestic consumption alone, causing elevated concentrations of various antibiotics in rivers across all continents. For example, estimated concentrations of antibiotics in 6 million km of rivers worldwide exceed thresholds that are protective of ecosystems and/or resistance promotion, with the most impacted regions located in Southeast Asia. As global antibiotic consumption rises, especially in low- and middle-income countries, these findings underscore the urgent need for the development and implementation of strategies to minimize antibiotic pollution and safeguard environmental and human health.

## Introduction

Studies over the last decade have reported substantial amounts of antibiotics in surface waters (1), drinking water (2), groundwater (3), and sediments (4). Antibiotics in natural water systems, even at chronic subtherapeutic concentrations, are of concern because they have been shown to reduce microbial diversity (5), increase the presence of resistance genes (6), and potentially impact the health of fish (7) and algae (8). The World Health Organization (WHO) has recognized antimicrobial resistance (AMR) as a critical global threat, with projections suggesting that resistant bacterial infections could become the leading cause of global deaths by 2050 (9, 10).

Antibiotics are released into the environment due to their incomplete metabolism in the human body and incomplete removal in wastewater systems (11); due to the extensive use of antibiotics in animal husbandry (12) and in the aquaculture industry (13); and due to losses during manufacturing processes (14). While pharmaceutical and aquaculture industries are often regulated with respect to permissible effluent contaminant loads, due to their point source nature, waste from animal husbandry is often dispersed, reaching surface waters through indirect pathways such as soil and groundwater (15, 16). Conversely, wastewaters from households and hospitals are commonly discharged directly into wastewater handling systems; thus, anthropogenic effluents represent important point sources of contaminants into receiving surface waters (17–19). In fact, the importance of anthropogenic sources has increased over time given that human consumption of antibiotics increased globally by 65% between 2000 and 2015, mostly driven by low-income countries that experienced an increase of 114% (20). Projections from the same study expect an increase of 200% by 2030. While some advanced treatment methods can substantially decrease or virtually eliminate antibiotic residues in wastewaters (21), such technologies have not been implemented at most municipal wastewater treatment plants (WWTPs). In effect, almost half of the world's wastewater is released to the environment without any treatment (22). Furthermore, part of the natural or artificial “removal” may simply involve partitioning to the solid phase (e.g. stream sediments) (23), or conversion into transformation products during metabolism or treatment, which can still exhibit antibiotic properties and retain toxicological significance (24, 25). In some cases, certain compounds can even revert to their parent form after environmental discharge (26).

Given the potential risks to the environment and human health due to antibiotics exposure, it is crucial to assess the prevalence of these compounds in wastewaters and surface waters globally. However, sensitive, complicated, and expensive analytical equipment and techniques are needed to detect or quantify antibiotics in situ and on a regular basis, especially when present in low concentrations, thereby severely limiting the scope and feasibility of regular monitoring (27). In the absence of adequate large-scale monitoring schemes, modeling approaches can provide an initial global outlook on the possible risks caused by a wide range of chemical substances, including antibiotics. In particular, contaminant fate models that simulate the use and fate of chemical substances in the aquatic environment can serve as a screening tool to pinpoint the most vulnerable regions that may require the implementation of new and improved environmental management strategies or regulations (28, 29).

Here, we use the global contaminant fate model HydroFATE (30) (see Methods for more details) to estimate the concentrations of the 40 most consumed antibiotics by humans (i.e. representing 90% of total worldwide human antibiotic consumption, see Table 1 for the list of antibiotics used in the study) (20) in the global river network by investigating 23.8 million km of total river length. Due to limited availability of consistent global effluent data related to antibiotics originating from veterinary/livestock and industrial sources (including manufacturing losses), our analysis encompasses only residual antibiotics originating from domestic sources, i.e. those that are consumed by the global human population and are released to rivers and lakes through wastewater treatment systems or through untreated pathways. Our focus is therefore on determining whether antibiotics that originate from human consumption represent a source of risk by themselves and, if so, how this risk can be mitigated or managed through appropriate strategies.

**Table 1.** Antibiotics selected for this study and their input parameters in HydroFATE.

	Substance	Annual global consumption (10^6^ kg)	Average excretion fraction (%)	Wastewater treatment removal efficiency (%)			Instream decay constant (day^−1^)	PNEC-ENV (ng L^−1^)	PNEC-MIC (ng L^−1^)	DDD(g)	ADI (µg kg^−1^ day^−1^)
				Primary	Secondary	Advanced					
**1**	**Amoxicillin**	11.8	71 (32)	0	85 (32)	100 (36)	2.6 (37, 38)	570	250	1.5	0.43
**2**	**Sulfamethoxazole**	2.0	28 (32)	0 (39, 40)	50 (32)	75.5 (41)	0.77 (42)	600	16,000	1.9	10
**3**	**Ciprofloxacin**	1.8	70 (32)	31.6 (43–46)	70 (32)	85.5 (41, 43)	1.59 (47, 48)	450	60	1	0.15
**4**	**Cefalexin**	1.3	92.5 (32)	0	81 (32)	81	0.017	210	4,000	2	10
**5**	**Ampicillin**	1.2	74 (32)	0	75 (32)	90 (41)	0.017	600	250	2	0.43
6	Cefuroxime	0.9	96.5 (32)	0.2	41	41	0.017	1,700	500	0.5	0.14
7	Piperacillin	0.7	82 (32)	0	80 (49)	83 (49)	0.017	4,300	500	14	4.3
**8**	**Ceftriaxone**	0.8	56.5 (32)	0	0	66.7 (50)	0.017	330	30	2	0.03
**9**	**Azithromycin**	0.7	62.2 (32)	21.5 (39)	38.1 (39, 46, 51)	90 (41)	5.36 (52)	30	250	0.3	0.71
10	Cefixime	0.7	100 (32)	0	10 (53)	10	0.017	600	60	0.4	5.7
**11**	**Levofloxacin**	0.6	92.5 (32)	21 (46)	42 (32)	42	0.13 (54)	1,520	250	0.5	0.14
**12**	**Clarithromycin**	0.5	31.5 (32)	20 (39, 46)	26 (32)	55 (36, 55)	2.22 (52)	250	250	0.5	1.4
13	Cefadroxil	0.6	91.5 (32)	0	0	100 (56)	0.017	140	2,000	2	1.4
14	Penicillin V	0.4	58 (32)	0	40 (32)	40	0.017	n.a.	60	2	0.43
**15**	**Erythromycin**	0.5	24 (32)	0 (39)	31 (32)	43 (41)	2.057 (42, 48, 57)	500	1,000	1	5
16	Cloxacillin	0.5	49.5 (58)	5	56	56	0.017	20,000	130	2	n.a
**17**	**Tetracycline**	0.5	100 (32)	22	76	90 (36)	4.32 (48)	3,200	1,000	1	3
**18**	**Trimethoprim**	0.4	66.5 (32)	19 (39, 45)	30 (32)	82.5 (41)	0.478 (47, 52, 54, 57)	312,450	500	0.4	4
**19**	**Clindamycin**	0.3	12.6 (32)	0 (32)	0 (32)	85 (36)	0.017	100	1,000	1.2	0.14
20	Cefazolin	0.3	91 (32)	0	67.5 (59)	67.5	0.017	n.a.	1,000	3	10
**21**	**Cefotaxime**	0.3	20 (58)	1	1	1	0.017	120	130	4	n.a.
22	Penicillin G	0.3	66 (32)	0.5	0.7	74.7 (60)	0.017	n.a.	250	3.6	0.43
23	Flucloxacillin	0.3	65.5 (61)	0 (62)	31 (62)	31	0.017	26,800	n.a.	2	n.a.
24	Cefpodoxime P.	0.3	32.5 (63)	5	67.5 (59)	67.5	0.017	1,760	250	0.4	n.a.
**25**	**Norfloxacin**	0.3	58 (32)	10.5 (43–45)	64 (32)	78 (41, 43)	1.466 (47, 48)	120,000	500	0.8	2.9
26	Cefaclor	0.3	60 (32)	75 (64)	96 (32)	96	0.017	n.a.	500	1	0.21
**27**	**Spiramycin**	0.3	4.4 (32)	0 (40)	5	9 (65)	0.017	1,090	500	3	50
**28**	**Doxycycline**	0.2	90 (32)	0 (32)	0 (32)	0	0.008 (66)	25,100	2,000	0.1	3
**29**	**Chloramphenicol**	0.2	98 (32)	0.9	93 (32)	100 (36)	0.017	n.a.	8,000	3	0.125
**30**	**Ofloxacin**	0.2	78 (32)	0 (44)	69 (32)	93.5 (41)	4.25 (48, 52)	10,000	500	0.4	5.7
31	Ceftazidime	0.2	87 (32)	0	0	0	0.017	1,300	500	4	0.21
**32**	**Roxithromycin**	0.1	36.5 (32)	13 (39, 46)	42 (32)	42	2.736 (48, 52)	6,800	1,000	0.3	2.1
**33**	**Lincomycin**	0.2	72.5 (67)	8 (68)	36 (46, 51, 68, 69)	50 (36)	0.017	810	2,000	1.8	n.a.
34	Amikacin	0.1	98.5 (58)	0	78	78	0.017	n.a.	16,000	1	n.a.
35	Meropenem	0.1	65 (58)	0	85 (36)	87 (36, 50)	0.017	1,500	60	3	n.a.
36	Moxifloxacin	0.1	45 (58)	0 (44)	64 (70)	64	0.017	n.a.	130	0.4	n.a.
37	Vancomycin	0.1	95 (32)	0 (32)	54.8 (32, 33, 71)	100 (36)	0.017	n.a.	8,000	2	1.4
38	Gentamicin	0.1	65 (32)	0	0	0	0.017	150	1,000	0.24	4
39	Cefepime	0.1	85 (72)	6	9	9	0.017	1,300	500	4	n.a.
40	Imipenem	<0.1	38 (58)	0	0	0	0.017	410	130	2	0.43

The annual global consumption represents the average for the period of 2012–2015 for each substance as provided by Klein et al. (20) aggregated worldwide, including the fraction of combination drugs and extrapolated values. Efficiency values in the “Primary” and “Secondary” treatment columns for which there were no available literature sources were estimated using the model SimpleTreat 4.0 (31). Most values of average excretion fraction and secondary treatment were compiled by Khan (32) based on literature sources. Values in the “Advanced” treatment column for which there were no available literature sources were assumed to be the same as the “Secondary” value. Values in the “Instream decay constant” column for which there were no available literature sources were assumed to be 0.017 day^−1^ based on the definition of a persistent chemical in the aquatic environment by the REACH regulation (33). When more than one value was reported by the same source or more than one source was available, an average value was used. PNEC-MIC and PNEC-ENV values were provided by AMR Industry Alliance (34). Defined daily dose (DDD) values were provided by WHO (35). Acceptable daily intake (ADI) values were provided by Khan and Nicell (2). Antibiotics with their name in bold were used in the model validation.

n.a., not available.

Estimates of initial substance emissions from domestic sources are based on population distribution, per capita consumption of the substance, and human metabolism. Removal of substances is then simulated depending on the contaminant pathway (i.e. through wastewater treatment or, if not treated, through direct discharge). The transport of the contaminants in the river system is simulated by accumulating the contaminant load downstream while accounting for instream decay and removal in lakes. Instream concentrations for every river reach are calculated by dividing the total combined contaminant load from upstream reaches plus local sources by the river discharge at the given location. Before application, the model was extensively validated by comparing simulated to reported concentrations of 21 antibiotics at 877 locations globally (see Methods). To portray a scenario of exposure when risk would be most prevalent yet within plausible limits, the default river discharge used in this analysis, unless stated otherwise, is the lowest monthly flow value within an average year (1971–2000) to represent low-flow conditions. Nonetheless, we do not consider this to be a true worst-case scenario as we refrain, due to limited data availability, from using (i) more extreme daily low flows and (ii) seasonal maxima or spikes in antibiotic consumption. This approach provides conservative estimates aiming for potential risks to be neither exaggerated nor underestimated, particularly in regions with substantial seasonal variations. We chose those settings after testing model outcomes by assuming various alternative scenarios representing upper and lower bounds of predicted concentrations (see Section S1 for a comprehensive evaluation of the model's performance).

The concentration estimates are then converted into proxy indicators representing the cumulative environmental and human health exposure to these antibiotics (see Methods). These indicators were not designed to be comprehensive; rather, they aim to portray key risks associated with antibiotic pollution, such as environmental toxicity and microbial resistance promotion (environmental exposure), and drinking water consumption (human health exposure). First, to assess environmental exposure, we calculate the individual risk quotient (RQ*_i_*) associated with each antibiotic, expressed as the ratio of the aquatic concentration estimated by the model divided by the lowest reported threshold of environmental impact, or of microbial resistance promotion, for that chemical. Different RQ*_i_* levels can be interpreted as representing different risk categories, here ranked as “low risk” (0.01 ≤RQ*_i_* < 0.1), “medium risk” (0.1 ≤ RQ*_i_* < 1), and “high risk” (RQ*_i_* ≥ 1) (73, 74).

In our multicompound assessment, we account for the cumulative exposure to multiple antibiotics by summing the RQ*_i_* values of individual substances into a total risk quotient, RQ_tot_, following the concentration addition concept of mixture toxicity (75–77). Since coexisting pollutants and mixture effects of multiple chemicals can have unforeseen impacts on aquatic ecosystems in a synergistic or antagonistic manner, i.e. amplifying or subduing the environmental risk (78, 79), concentration addition is considered a justifiable approach for preliminary risk assessments (80). As was done with RQ*_i_*, a mixture of chemicals presenting RQ_tot_ ≥ 1 is assumed to pose a potential risk (81). Here, we apply the same risk categorization for RQ_tot_ as we did for RQ*_i_*, i.e. low risk (0.01 ≤ RQ_tot_ < 0.1), medium risk (0.1 ≤ RQ_tot_ < 1), and high risk (1 ≤ RQ_tot_ < 10), but extended to include a “very high risk” category (RQ_tot_ ≥ 10).

Second, to assess human health exposure, we calculate the equivalent dose concentration for each compound, which is defined as the ratio of the aquatic concentration predicted by the model relative to the defined daily dose (DDD) of the antibiotic (see Methods for details). The values for individual substances are subsequently aggregated to estimate the cumulative equivalent antibiotic dose concentration for each river reach. After defining the 99th percentile as a proxy for high levels of antibiotic dose concentration in the river network, we calculate the predicted daily intake of antibiotics at this concentration level by assuming the surface water is directly used for human consumption; i.e. without removal via drinking water treatment processes. This value is then compared to available thresholds of chronic exposure to selected antibiotics to assess if there is a potential human health risk in these rivers presenting high dose concentrations (74).

## Results

### Emission of antibiotics to the world's inland surface waters

**Fig. 1.**
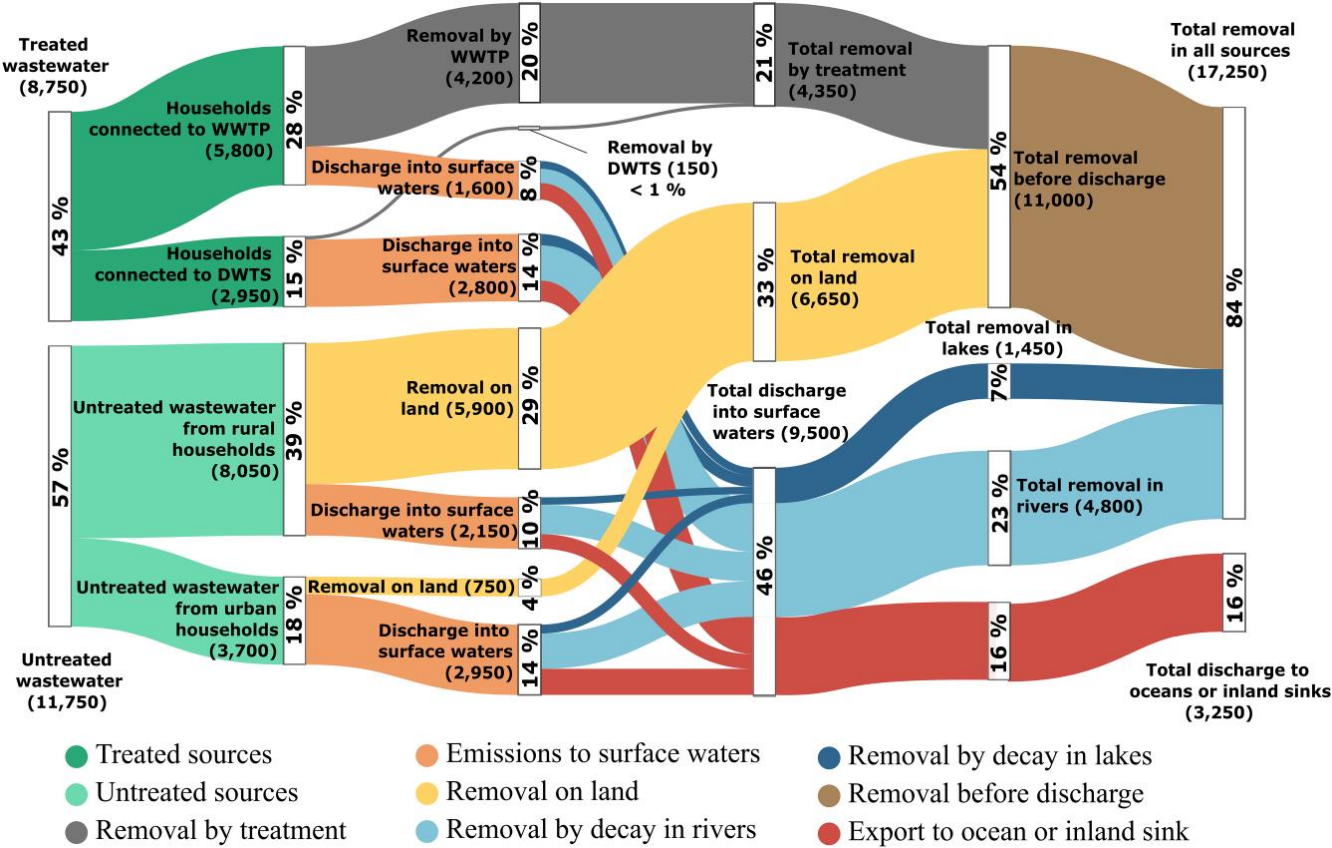
Contaminant pathways of antibiotics in the global aquatic environment. Modeled contaminant pathways and mass balances of antibiotics by path. Values in parentheses indicate total amounts of the top 40 antibiotics consumed worldwide in tonnes year^−1^; percentage values are relative to the total excretion amount (20,500 tonnes year^−1^).

The total global human consumption of antibiotics is estimated at 32,200 tonnes year^−1^ based on data for 2012–2015 (20), of which the 40 antibiotics that are most used by the world's population represent a combined 29,200 tonnes year^−1^ (91%). Including the extrapolated consumption from countries not included in the sales data (see Global consumption of antibiotics section in Methods for more details), this number increases to 30,300 tonnes year^−1^. By differentiating the various pathways of antibiotics in the aquatic environment (Fig. 1), we estimate that, from this combined consumption, 68% (20,500 tonnes year^−1^) are excreted after metabolism, 31% (9,500 tonnes year^−1^) are discharged into surface waters after treatment or natural attenuation in soils, and 11% (3,250 tonnes year^−1^) reach the world's oceans or inland sinks through rivers after lake removal and instream decay. Note that “Removal on land” in Fig. 1 represents only the amounts of antibiotics that did not reach surface waters due to natural attenuation processes in soils affecting wastewater that has not undergone treatment. It does not include land-applied sludge from wastewater treatment processes, which were not considered in this study.

We estimate that only 43% of the load of antibiotics from domestic sources is processed in wastewater systems, while households without access to wastewater treatment represent 39 and 18% of total emissions from rural and urban areas, respectively. On average, centralized WWTPs are estimated to remove 72% of the total antibiotic load reaching all facilities and in turn contribute 17% of the total emission of antibiotics into surface waters. Decentralized wastewater systems, due to the low average removal efficiency of 5%, contribute 29% of the total emission into rivers. The combined untreated domestic wastewaters from rural and urban areas contribute the greatest share of total emissions into rivers with 23 and 31%, respectively. After the introduction of the antibiotics into surface waters, instream decay is the dominant removal process (48%), and lakes and reservoirs remove another 13%.

### Environmental exposure to antibiotic residues in rivers

**Fig. 2.**
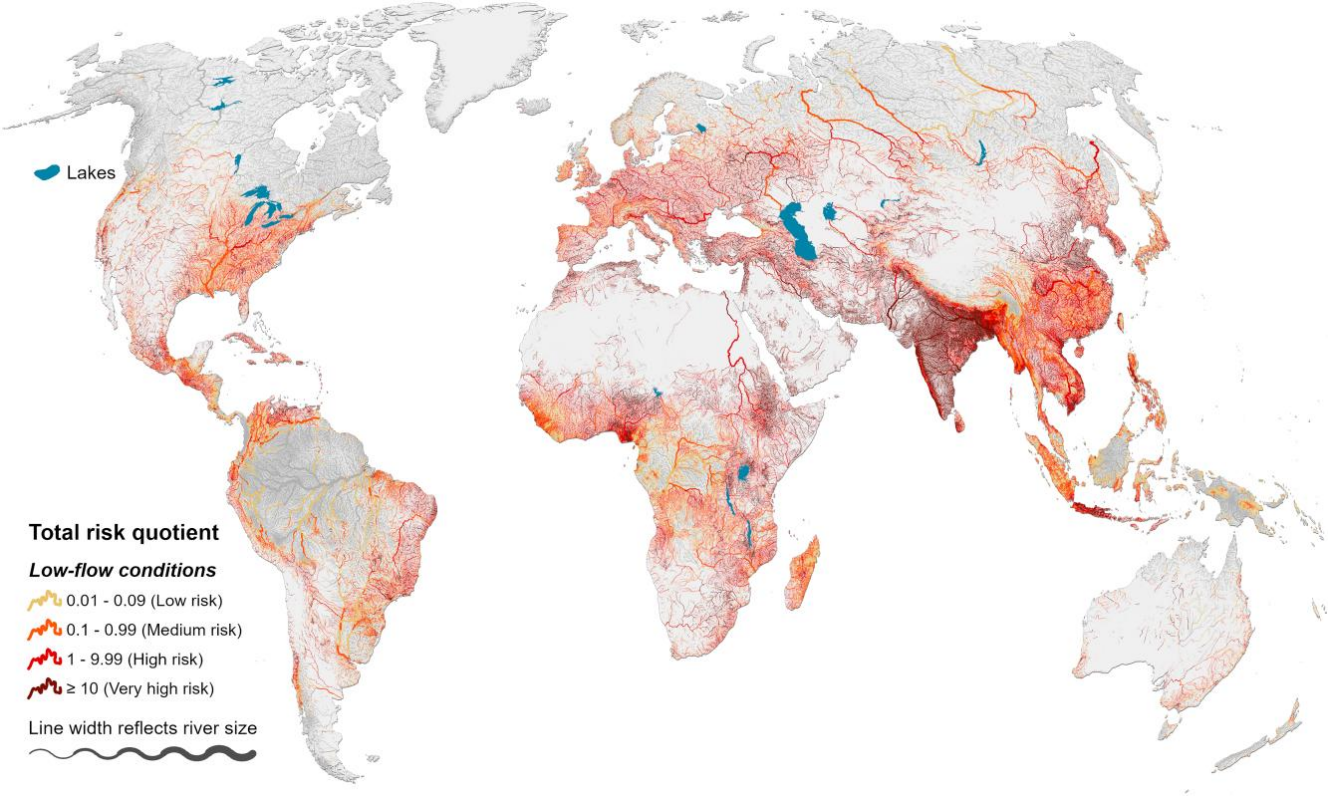
Environmental exposure levels of antibiotics in global rivers. Total risk quotient (RQ_tot_) is calculated as the sum of individual risk quotients of all 40 antibiotics in the global river system under low-flow conditions. Rivers in gray present insignificant risk (RQ_tot_ < 0.01). Only rivers exceeding a long-term average flow of 0.1 m^3^ s^−1^ are shown.

At low-flow conditions, 49% of the total investigated river length (23.8 million km) fall below any of the assessed levels of potential environmental impact or antibiotic resistance when analyzing the combined risk quotient of the 40 most used domestic antibiotics (RQ_tot_ < 0.01), 9% of river length registers at the low risk level (0.01 ≤ RQ_tot_ < 0.1), and 17% at the medium risk level (0.1 ≤ RQ_tot_ < 0.99) (Fig. 2). For 6.0 million km of rivers (25%), the combined risk quotient exceeds the threshold of high risk (i.e. RQ_tot_ ≥ 1), with 2.2 million km surpassing risk levels deemed to be very high (RQ_tot_ ≥ 10) (Table 2 and Fig. 2). In 3.8 million km of rivers, at least one substance exceeds an RQ*_i_* of 1 (RQ_max_ ≥ 1 in Table 2), and 0.7 million km of rivers are affected by at least 10 substances that each exceed an RQ*_i_* of 1.

**Table 2.** Length of rivers presenting high environmental risk from antibiotic concentrations at low-flow conditions.

Country	*Environmental exposure*							*Human health exposure*		
	RQ_tot_ ≥ 1		RQ_max_ ≥ 1			RQ*_i_* ≥ 1 for at least 10 substances		*D*_eq_ ≥ 99th percentile		
	Length of rivers (10^3^ km)	% of total	Length of rivers (10^3^ km)	% of total	Most contributing substance	Length of rivers (10^3^ km)	% of total	Length of rivers (10^3^ km)	% of total	Population exposed (millions)^a^
India	677.0	87.2	623.0	80.3	Cefixime	274.0	35.3	111.0	14.3	315.0
China	669.0	46.4	353.0	24.5	Amoxicillin	74.4	5.2	24.6	1.7	89.8
Russia	285.0	8.3	217.0	6.3	Ceftriaxone	7.2	0.2	3.2	0.1	11.2
United States	260.0	14.6	97.6	5.5	Amoxicillin	13.2	0.7	7.2	0.4	11.8
Brazil	211.0	8.8	109.0	4.5	Ciprofloxacin	7.2	0.3	3.7	0.2	33.9
Iran^b^	183.0	90.7	147.0	72.7	Amoxicillin	29.4	14.5	10.8	5.3	9.3
Nigeria^b^	174.0	86.2	120.0	59.5	Amoxicillin	15.7	7.8	4.7	2.3	18.2
Ethiopia^b^	162.0	87.4	125.0	67.3	Amoxicillin	18.7	10.1	5.4	2.9	7.2
Turkey	127.0	84.0	93.7	61.9	Amoxicillin	12.9	8.5	8.9	5.9	16.2
DR Congo^b^	102.0	18.4	33.2	6.0	Amoxicillin	1.2	0.2	0.4	0.1	7.6
Indonesia	101.0	14.4	62.7	8.9	Amoxicillin	1.6	0.2	0.4	0.1	5.0
Thailand	97.0	71.6	64.7	47.8	Amoxicillin	2.0	1.4	0.9	0.7	8.2
Vietnam	96.5	88.0	52.1	47.5	Cefalexin	12.8	11.7	2.1	2.0	16.9
Mexico	95.2	35.2	50.5	18.7	Ciprofloxacin	9.2	3.4	4.5	1.6	12.3
Pakistan	91.2	89.8	77.6	76.4	Ciprofloxacin	31.4	31.0	17.2	17.0	23.0
Total	3,330.0	26.8	2,230.0	17.9	Amoxicillin	511.0	4.1	205.0	1.6	586.0
Global	5,980.0	25.1	3,810.0	16.0	Amoxicillin	723.0	3.0	324.0	1.4	751.0
**River discharge (m^3^** **s^−1^)**										
10,000–100,000	3.1	12.8	0.8	3.3	Cefixime	0.0	0.0	0.0	0.0	0.0
1,000–10,000	37.8	24.0	20.2	12.9	Cefixime	0.7	0.4	0.0	0.0	0.0
100–1,000	178.0	28.0	105.0	16.5	Ceftriaxone	7.7	1.2	1.3	0.2	4.0
10–100	548.0	29.2	341.0	18.2	Ceftriaxone	54.6	2.9	16.4	0.9	29.6
1–10	1,500.0	24.4	933.0	15.2	Amoxicillin	180.0	2.9	77.6	1.3	150.0
0.1–1	3,720.0	24.7	2,410.0	16.1	Amoxicillin	480.0	3.2	228.0	1.5	568.0
Total	5,980.0	25.1	3,810.0	16.0	Amoxicillin	723.0	3.0	324.0	1.4	751.0

Data shown are for top 15 countries (for a complete list of all countries, see Table S2) and for different river size categories. The size classification is based on long-term (1971–2000) average flows, but the exposure analysis is conducted at low-flow conditions. Note that very large rivers exceeding 100,000 m^3^ s^−1^, i.e. the mainstem Amazon, did not show risk. “RQ_tot_ ≥ 1” accounts for all river reaches where the sum of individual risk quotients for all 40 substances equals or exceeds 1; “RQ_max_ ≥ 1” accounts for all river reaches where the individual RQ of at least one substance equals or exceeds 1; “*D*_eq_ ≥ 99th percentile” accounts for those river reaches that represent the 99th percentile with highest cumulative equivalent dose concentration(i.e. 29 µD_eq_ L^−1^; see explanations in text). “% of total” represents the percentage of length compared with all rivers in the national or global river network, respectively, exceeding a long-term average flow of 0.1 m^3^ s^−1^ (23.8 million km).

^a^Estimated as those living within 10 km from a river reach that exceeds 29 µD_eq_ L^−1^.

^b^Countries for which the antibiotic consumption was not reported but estimated by extrapolation (see Methods).

From the 15 countries with the highest total length of rivers in the high or very high risk categories (i.e. RQ_tot_ ≥ 1) at low-flow conditions (Table 2), India, Iran, Nigeria, Ethiopia, Turkey, Vietnam, and Pakistan have more than 80% of their respective rivers in these categories. In addition to the concentration of antibiotics, a variety of substances present similar patterns of spatial variability. For example, in India and Pakistan, more than 30% of rivers are impacted by at least 10 antibiotics with aquatic concentrations that surpass their individual high-risk thresholds at low-flow conditions (Table 2). These results are corroborated by measurements reported in the literature (27, 53, 82).

Finally, we find that a total of 5.0 million km of rivers deemed to be at high environmental risk (including 1.8 million km of rivers at very high risk) represent rivers that are currently considered to be free-flowing (83), i.e. rivers that are not substantially impacted by human activities altering their connectivity and ecosystem services. Thus, contamination is a principal risk factor to these otherwise intact rivers.

### Antibiotics of particular concern

**Fig. 3.**
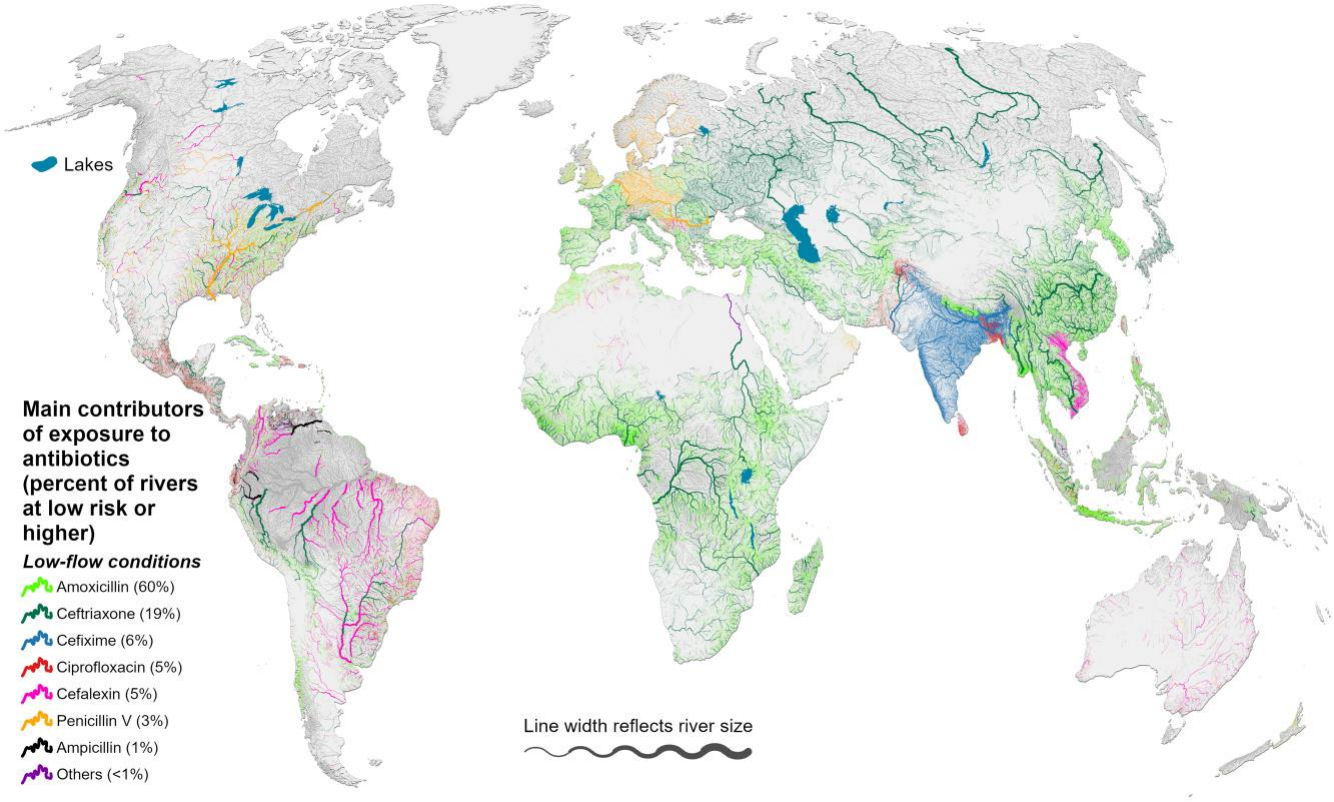
Antibiotics representing the main contributors of environmental exposure. Rivers in color depict those where at least one substance individually presents low risk (RQ_max_ ≥ 0.01) or higher under low-flow conditions, totaling 12.2 million km. Rivers in gray present insignificant risk (RQ_max_ < 0.01). Only rivers exceeding a long-term average flow of 0.1 m^3^ s^−1^ are shown on the map.

Among the 3.8 million km of rivers that have at least one substance presenting high environmental risk at low-flow conditions, the main compounds contributing to these high exposure levels are amoxicillin (45% by length), ceftriaxone (25%), and cefixime (17%). Figure 3 shows the results for rivers where at least one substance presents low risk. Amoxicillin, from the penicillin group, is expected to pose a higher risk in smaller streams (Table 2 and Fig. 3) and closer to the effluent sources due to its susceptibility to instream decay. Of all antibiotics, it has by far the highest global consumption (i.e. 75% of the total as reported by Klein et al. (20) as it is easily accessed and often available without prescription (28). Yet, reports of the detection of amoxicillin in environmental water samples are rare. This may be because most analytical methods are incapable of detecting penicillins, due to the instability of the β-lactam ring which is susceptible to hydrolysis, biodegradation, photolysis, and other decay processes (27, 37). Nevertheless, amoxicillin has been detected in surface water before (84–86), and considering its high use, it is important to trace its sources, especially because transformation products and resistance genes deriving from its metabolism processes can also affect the environment and human health (37, 87, 88).

Where amoxicillin is not the main contributor of exposure, other β-lactam antibiotics stand out, especially cefixime and ceftriaxone. In the three categories developed by the WHO in 2017 to guide the use of antibiotics in order to minimize potential AMR—Access, Watch, and Reserve—these two antibiotics are both classified in the “Watch Group,” whereas amoxicillin is classified in the “Access Group.” Watch Group antibiotics have high resistance potential when compared with the Access Group and should be used with more caution (89). However, there is a global increase in the consumption of Watch Group antibiotics, largely driven by India and China (28). For example, the cost of generic amoxicillin is sometimes greater than newer Watch Group drugs in India (90), a country already facing antibiotic overuse in human medicine and an extremely high (and increasing) prevalence of antibiotic-resistant bacteria (91). Cefixime is the primary contributor of exposure (RQ_max_ ≥ 1) in more than 80% of rivers in India.

Despite their high levels of reported consumption, it should be noted that for cefixime and ceftriaxone, there is a notable lack of information about their removal during waste treatment and their potential decay in surface waters. As a result, due to the assumptions made in the present study, downstream concentrations for these compounds may have been overestimated (see Methods for assumptions). In general, there is a concerning lack of robust and reliable ecotoxicity data for most pharmaceuticals (92), with assessments typically focusing on just a few selected substances, most of which do not rank high with respect to the risks they pose to aquatic organisms (93). The results presented here suggest an urgent need for studies on the environmental fate and impact of those antibiotics that are predicted to dominate in surface waters, and particularly those which pose a high potential risk.

### Human health exposure and risks

We estimate that 324,000 km of rivers surpass 29 microdose equivalents per liter (µd_eq_ L^−1^), which represents the 99th percentile of river reaches with highest equivalent dose concentrations (Table 2). The location of these rivers generally coincides with areas of highest environmental risk (compare Fig. 2). While equivalent dose concentrations at the microlevel may appear low, the consumption expressed in dose equivalents per liter is based on the DDDs of antibiotics, which reflect the therapeutic levels of the drug (35). Effects of chronic exposure to low concentrations of antibiotics are uncertain. The most likely risk associated with chronic exposure to antibiotic residues is resistance selection for human commensal bacteria, especially in the intestinal flora (94), but there have also been associations with allergic, autoimmune, metabolic, and psychiatric diseases, as well as colon cancer (95). This risk is best expressed in terms of the acceptable daily intake (ADI).

To assess the correlation between high exposure levels (99th percentile), equivalent dose concentrations, and potential health risk (exceedance of ADIs), we assume that an average-weighted person (60 kg) consumes 2 L day^−1^ of drinking water and that this amount is directly taken from surface waters without further treatment. If we further assume that the total equivalent dose concentration of the 99th percentile (29 µD_eq_ L^−1^) is associated with only one particular antibiotic, we can use the antibiotic's specific DDD to calculate the resulting daily intake of that particular substance. For example, the predicted daily intake of the antibiotic ciprofloxacin at 2 L day^−1^ drinking water consumption from a river reach that exceeds the 99th percentile equivalent dose concentration would amount to 0.97 µg kg^−1^ (see Methods). This dose surpasses the ADI for ciprofloxacin (96) (0.15 µg kg^−1^), indicating that the 99th percentile equivalent dose concentration may represent a risk to human health if exceeded by ciprofloxacin. Note that ciprofloxacin is also indicated as a main contributor of environmental risk (Fig. 3) and is one of the top three most consumed antibiotics worldwide (see Methods). In addition, the comprehensive study by Wilkinson et al. (1) reports at least 10 measurements of ciprofloxacin above 29 µg L^−1^ in different regions of the world, demonstrating that these high concentrations have been detected in surface waters. When the same approach is used to express equivalents in terms of the 29 other substances for which ADIs have been reported, 15 of these result in estimated daily intakes that exceed their respective ADIs (see Methods). We therefore consider the 99th percentile of highest equivalent dose concentrations to be a meaningful first-order proxy to highlight river reaches that pose a potential human health risk.

We estimate that 750 million people live in relatively close proximity (≤10 km) to these rivers, suggesting that 10% of the global population is exposed to the top 1% of surface waters with the highest cumulative concentrations of antibiotics. These populations are thus potentially subjected to chronic antibiotic intake at harmful levels if surface waters are used for direct human consumption. These numbers should be interpreted with caution, as they are based on assumptions and estimations (see uncertainties related to these results in Human health risk assessment section in Methods) that were necessary in light of current limited availability of data. As such, our predictions should primarily be used to identify regions on which subsequent research should be focused. For example, even if the United States shows areas with high concentrations and appears to have 11.8 million people at risk, it should be noted that the country is known to have various advanced programs in place for wastewater and drinking water treatment, as well as for monitoring emerging contaminants (97). This may also be the case in European countries (98), where the risk and population affected might be overestimated due to additional moderating processes not considered in this analysis. Nevertheless, antibiotics have been found in drinking waters after treatment in as the United States, Korea, China, and Iraq (99–102). Beyond toxicity from drinking water, pathogens developing resistance and causing antibiotic-resistant infections can affect people not only through drinking but also through other activities, such as bathing, laundry, and irrigation (103, 104). A preliminary global assessment is therefore crucial to understand the various levels and extents of potential exposure for populations and aquatic ecosystems to antibiotics in global surface waters. This assessment can also drive further investigation into diverse exposure pathways as more data become available.

India, China, and Pakistan hold 47% of the rivers with highest equivalent dose concentrations (i.e. 99th percentile). These are densely populated areas where signs of increasing bacterial resistance have been reported (20, 105, 106). For example, Murray et al. (107) estimated that 2.4 million deaths in 2019 were associated with bacterial resistance in South Asia, Southeast Asia, East Asia, and Oceania, representing 49% of the global estimation (4.95 million deaths).

## Discussion

### Exacerbating factors for risks from antibiotics

The results indicate that the contaminant loads that are associated with direct human consumption of antibiotics represent a considerable source of risk to aquatic ecosystems and human health. Importantly, the surface water concentrations predicted here may not reflect circumstances that may lead to much higher concentrations, and hence greater risks, in certain streams and under certain conditions that may arise over time.

For instance, global consumption of antibiotics in food animal production was estimated at 63,151 tonnes in 2010 (108) (i.e. about 2-fold the domestic consumption assumed in this study for the period of average 2012 to 2015) and the use of antibiotics in the aquaculture industry was estimated at 10,259 tonnes in 2017 (13). All seven antibiotics identified in Fig. 3 as the main contributors to risk arising from human consumption are also included in the list of antimicrobial agents of veterinary importance (109), i.e. they are also authorized for use in food-producing animals. These might contribute to surface water contamination in regions where animal husbandry practices are common. However, it is important to note that the pathways of veterinary antibiotics from point of use to surface waters are even more uncertain than those of human antibiotics. Point source pollution from veterinary sources can occur, such as from concentrated animal feeding operations (110), from meat packing facilities (111), or from rapid transport episodes (e.g. manure spills, streams where livestock have direct access to excrete directly into the stream, or areas with tile drains that short circuit the natural transport pathway) (112–114). But the pathways from dispersed animal excretions often involve soil dynamics that provide more time for decay processes to occur before subsurface runoff or groundwater baseflow indirectly transport the substances back to surface waters (15, 16), leading to reduced loads into surface waters from those sources. For example, one modeling study estimated that <1% of administered veterinary antibiotics reach surface waters (115). This contrasts with our estimates suggesting that 10% of antibiotics consumed by humans arrive at surface waters. Nevertheless, with large quantities of veterinary antibiotics being used in concentrated areas or operations, it is likely that some specific river reaches will be subject to significantly greater antibiotic loads than those estimated here.

Furthermore, municipal wastewater facilities frequently receive waste inputs from the pharmaceutical industry (116), which are not accounted for in the total antibiotic loads into WWTPs estimated in this study. Additionally, manufacturers may be significant point sources of contaminants when effluents containing residual antibiotics are released, either treated or untreated, into surface waters nearby (117). While the release of pollutants from industries is often regulated, there is lack of policies and regulations in place that adequately address antibiotic pollution and related risks from the pharmaceutical manufacturing sector (118). Moreover, compliance with respect to regulatory standards for effluent quality is inconsistent, even in high-income countries (119).

Consequently, total loads of antibiotics into surface waters are expected to be significantly higher than estimates predicted here for those localities where there are significant levels of antibiotic manufacturing or animal husbandry activities. As such, these specific areas of potential high risk would not be captured in this study. Of course, while it would be desirable to account for these other sources of antibiotic contamination, the modeling of such sources is not currently possible due to the lack of data both globally and regionally with respect to local manufacturing and animal husbandry practices, waste management practices, and characteristics that govern the transport, degradation, and emissions of antibiotic residues into surface waters. Nevertheless, by establishing a baseline level of contamination arising from human consumption, we aim to pave the way for more comprehensive studies in the future, including the modeling of other antibiotic sources.

Finally, the model design does not consider short-term peak concentrations which may be caused by seasonal fluctuations in the consumption or release of substances into the river network or by increased concentrations during extended dry periods with less dilution potential (17). For example, consumption rates in the period of 2000 to 2010 peaked between January and March for the northern hemisphere, between July and November for the southern hemisphere, and, as an exception, between July and September in India, coinciding with the end of the monsoon season (120). The model does not consider seasonality for both hydrologic conditions and antibiotic consumption mostly due to the absence of consumption data at a higher temporal resolution. Beyond this, it should be noted that our current model is designed to assess long-term, continuous releases and is not structured to capture episodic contamination events (e.g. sudden short-term releases of high contaminant loads, periods of very high consumption of antibiotics, such as during a pandemic, etc.). As such, there may be temporal variations in antibiotic concentration that could represent significant short-term risk in certain localities.

Despite its limitations, our evaluation of the model's predictive abilities against field measurements (see Section S1) indicates that it performs well under average conditions. This supports a critically important finding of this study; that is, direct consumption of antibiotics by humans alone poses a significant risk to river reaches in many areas of the world. This suggests that the development of strategies to manage antibiotic contamination arising from human consumption—and other sources including animal husbandry and manufacturing—is warranted. This is especially important given that antibiotic consumption is expected to increase globally (20), particularly in low- and middle-income countries, which will lead to higher antibiotic residue concentrations in rivers. Furthermore, global environmental change, both driven by climate change and water use alterations, is likely to lead to more extreme low-flow conditions at certain times in affected areas, which would exacerbate risks associated with antibiotic contamination (121).

### Implications for policy and practice

The findings of this study highlight the need to develop and implement appropriate wastewater management plans, upgrade existing wastewater treatment practices and facilities, and improve regulatory guidelines with a particular focus on high-risk substances and locations that pose the greatest risk. Our global model predicts high antibiotic concentrations in regions already impacted by antibiotic resistance, such as Southeast and South Asia (105, 107). Areas that exhibit the most widespread distribution of exposed river courses are those with a high population density and high antibiotic consumption and coincide with regions that offer only poor access to wastewater treatment and/or are subject to water scarcity (i.e. causing a lack of dilution in receiving waters). Expanding access to wastewater treatment facilities to areas of the world currently without such services would generally improve the quality of surface waters that receive waste effluents. This would not only reduce the prevalence of disease but also decrease the demand for antibiotics to treat diseases arising from contaminated water supplies.

We observe that most high-risk areas tend to coincide with regions of the world where antibiotics are readily available without prescription and are sometimes used prophylactically (122, 123). As such, improved healthcare practices that ensure the appropriate use of antibiotics are recommended. Concurrently, we recommend that the approval of new antibiotic drugs take a OneHealth approach that takes into account the potential environmental distribution based on modeled expectations before market release. Thus, regulatory bodies should preferentially approve those substances with screened concentrations below any given risk thresholds and favor the development and use of alternative substances with accelerated decay in the environment.

Given the uncertainties inherent in our global modeling effort, the results are not intended to be used as locally explicit risk predictions. Rather, our global exposure maps provide baseline information for the development of targeted local monitoring and impact assessment studies in regions of highest exposure. Additionally, it is critical to enhance our understanding of the pathways through which untreated wastewater is discharged into surface waters and of the decay of substances in the aquatic environment. Given the absence of established thresholds and common regulatory guidelines, more research on the limits of long-term human exposure to antibiotics is critically required to improve risk predictions. Finally, antibiotics releases from animal treatments, production facilities, and health centers should be added to the simulations which are expected to particularly exacerbate problems in livestock-intensive areas. A concerted implementation of these strategies is urgently needed to succeed in the common goal of safeguarding the health of ecosystems and human populations who vitally depend on high-quality water.

## Methods

### Overview of study design and model implementation

**Fig. 4.**
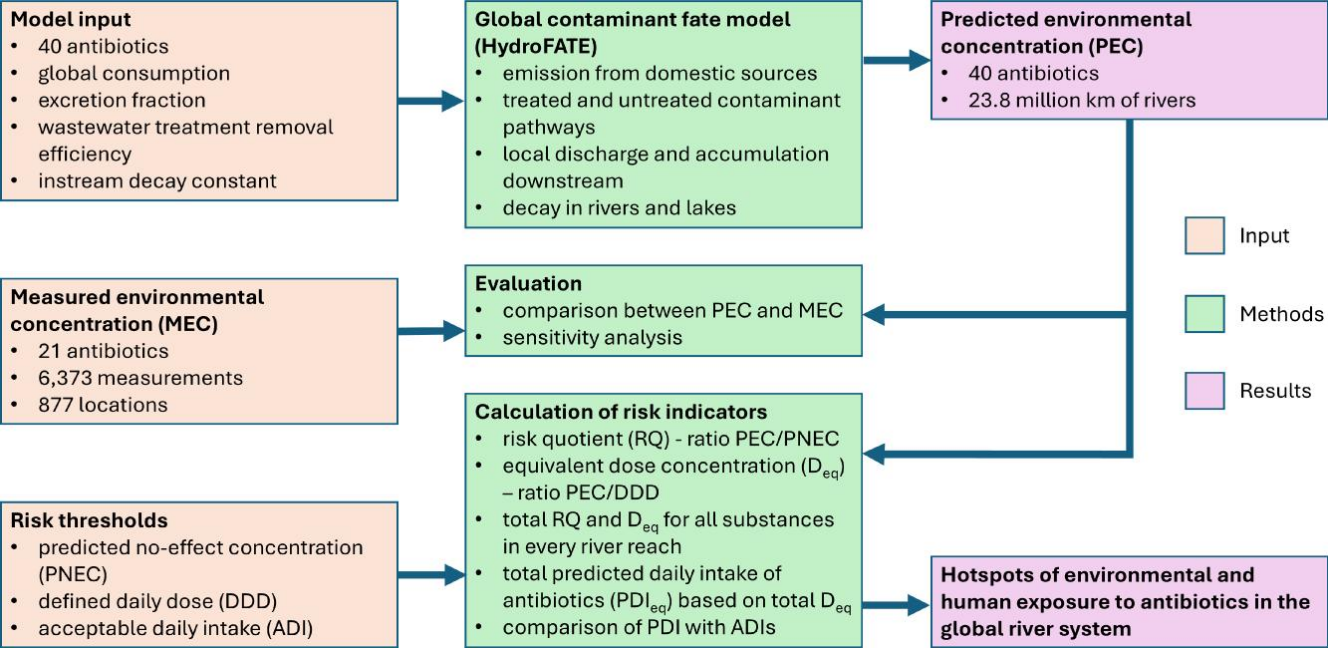
Overview of study design. For additional explanations, see text.

See Fig. 4 for a summary of the data and methods used in this study.

The components of HydroFATE are described in detail in previous publications in which the application of the model was demonstrated at regional (124, 125) and global scales (30). In summary, HydroFATE simulates the emission of a contaminant from households following consumption, its pathway through different treatment options (treated in centralized or decentralized wastewater facilities at various levels, or not treated) and subsequent release into surface waters, and finally its transport downstream. Estimates of initial substance emissions are based on population distribution, per capita consumption of the substance, and human metabolism. Removal of substances through wastewater treatment or natural attenuation in soils or subsurface flow is then simulated depending on the contaminant pathway. The emissions from populations served by a WWTP, or by a smaller and decentralized wastewater treatment system (DWTS), are reduced by a treatment removal efficiency based on the level of treatment offered by the facility. Emissions arising from those populations that are not served by any type of wastewater treatment are attenuated by a direct discharge coefficient that depends on whether the emission takes place in a rural area (further modulated by the distance from the river network) or urban area.

The contaminant loads from all pathways inside the incremental catchment boundaries of an individual river reach are aggregated to calculate the total local contaminant load of the reach. HydroFATE then employs the generic river routing model HydroROUT (83, 126, 127) to simulate the transport of chemical substances in the river system, accumulating the contaminant load downstream while accounting for instream decay (i.e. using a first-order decay function) and removal in lakes (i.e. using the “completely stirred reactors'” method for modeling lakes), both processes affected by river discharge (i.e. under average-flow or low-flow conditions). Finally, the predicted environmental concentration (PEC) for each contaminant is calculated by dividing the total local contaminant load plus the incoming load from upstream reaches by the river discharge in every river reach.

### River and lake network

The various raster and vector layers representing the river network and catchment boundaries in HydroFATE were obtained from a derivative of the HydroSHEDS database (128) known as RiverATLAS (129). The global river network of RiverATLAS contains 8,477,883 individual river reaches, with an average length of 4.2 km, representing a total of 35.9 million km of rivers globally. Each river reach has an associated catchment with an average area of 15.7 km^2^. The vectorized river network was extracted at 15-arcsecond (∼500 m at the equator) grid cell resolution and represents all rivers and streams where the average discharge exceeds 100 L s^−1^ or the upstream catchment area exceeds 10 km^2^. While HydroFATE runs for the whole river network, we only used those 23.8 million km of rivers whose average discharge exceeds 100 L s^−1^ to present and evaluate the results, in order to avoid potential artifacts at minor streams for which the flow estimates (derived from a global hydrological model) are increasingly uncertain and error-prone.

Each river reach in RiverATLAS is associated with a series of precalculated hydroenvironmental characteristics. From this database, long-term (i.e. 1971–2000) average naturalized river discharge estimates are used in HydroFATE. These estimates were derived from the global hydrological model WaterGAP version 2.2 (130) and were downscaled from their original resolution of 0.5° grid cells to the RiverATLAS resolution of 500 m using geostatistical techniques (127). In addition to annual average discharge estimates, minimum discharge estimates (i.e. the lowest monthly flow value within an average year) are available as a proxy for low-flow conditions (127).

To account for lake decay processes, HydroLAKES, a global database that provides the shoreline polygons of 1.4 million lakes with a surface area of at least 10 ha (131), is integrated in HydroFATE.

### Domestic sources and pathways of antibiotic residues

Six contaminant pathways are accounted for in the HydroFATE model as follows: point sources of treated wastewater from populations connected to WWTPs that provide (i) primary level of treatment, (ii) secondary level of treatment, or (iii) advanced (i.e. tertiary or higher) level of treatment; decentralized sources of treated wastewater from populations not connected to a WWTP but served by (iv) a DWTS; or diffuse sources of untreated wastewater from populations in (v) urban areas or (vi) rural areas. These pathways were incorporated into the HydroFATE model using the global WWTP database HydroWASTE (132), a population grid (133), an urban extent grid (134), and country-level sanitation statistics (135), as described in detail in Ehalt Macedo et al. (30).

### Contaminant data and parameter settings

#### Global consumption of antibiotics

**Fig. 5.**
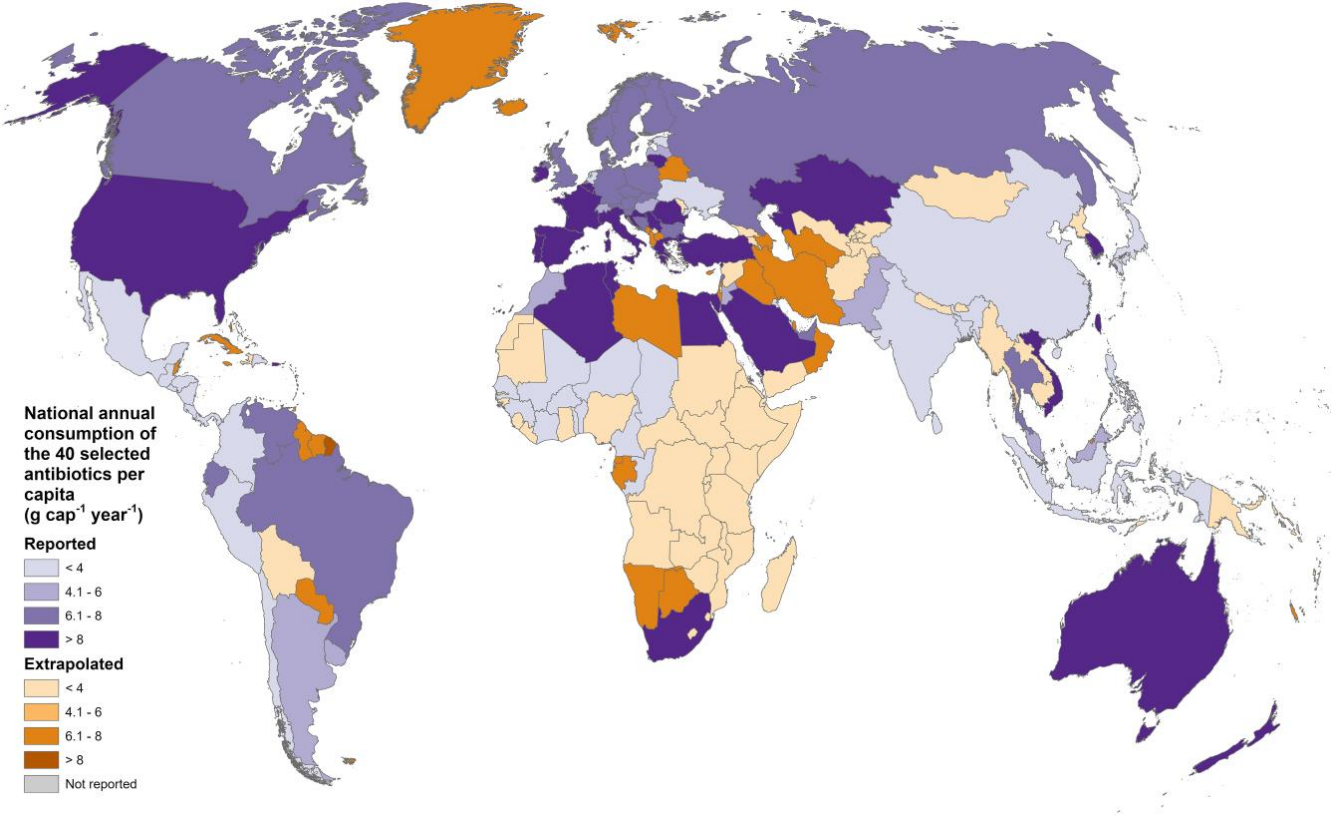
Annual consumption of the 40 antibiotics selected for inclusion in this study, aggregated by country. Reported values are from Klein et al. (20), and extrapolated values were calculated based on the methodology described in text.

Klein et al. (20) analyzed and estimated the consumption of antibiotics in the world based on the IQVIA MIDAS database, which reports annual sales for the period of 2012 to 2015 for 91 countries. In total during that period, 128,700 tonnes of 229 antibiotics, including combinations of more than one antibiotic used together and antibiotics used in conjunction with other substances, were estimated to have been consumed. The 40 antibiotics included in this study represent 90% of the mass of total worldwide antibiotic consumption for the four years from 2012 to 2015 (i.e. 117,000 tonnes) and were consumed in more than 50% of countries in the database (Table 1). We used the average annual consumption over 2012 to 2015 to calculate the annual per capita consumption based on the country's population in 2015. For the 17% of the global population whose consumption of antibiotics was not included in the database (see Fig. 5), annual consumption data were estimated based on the average per capita antibiotic use from populations in the same income group (136) following the methodology described by Klein et al. (20). For substances reported to be used in combination with other drugs (i.e. cases where two or more active substances are included in a single dosage, such as amoxicillin/clavulanic acid or sulfamethoxazole/trimethoprim), the portion of each relevant substance was added to their total consumption. The partition of combination drugs was based on their most common formulation.

The IQVIA MIDAS database has uncertainties that may affect the results of our study, particularly: (i) calculated use data are based on estimated sales data, which might not reliably reflect actual consumption including informal or illegal production (137); (ii) country-aggregated data do not account for local variability; and (iii) the data have not been validated particularly in low- and middle-income countries (20). Despite these limitations, to the best of our knowledge, the database provides the only source of harmonized data on global antibiotic consumption and the limitations likely fall within the margin of error of the model calculations.

#### Additional substance-specific parameters

Additional parameters that affect the final concentration of antibiotics simulated by HydroFATE are as follows: (i) the excretion fraction, reported as the fraction of the antibiotic that is excreted after human consumption and metabolism; (ii) the wastewater treatment removal efficiency, which is the estimated percentage of the inflowing contaminant load that is removed via wastewater treatment; and (iii) the instream first-order decay constant (i.e. corresponding to the natural logarithm of 2 divided by the half-life of the substance) that dictates the rate of decrease in contaminant concentration due to processes such as biodegradation or photodegradation during its transport along rivers and lakes. These parameters and their literature sources are listed in Table 1.

### Calculation of environmental and human exposure to antibiotics

The following parameters were compiled and/or calculated for the antibiotics in this study, and the values are reported in Table 1.

#### Predicted no-effect concentration

The predicted no-effect concentration (PNEC) is used as a protective threshold in environmental exposure assessments (138, 139). That is, when the concentration of a substance exceeds the PNEC, some measurable aquatic ecosystem impact may occur. For antibiotics, the AMR Industry Alliance periodically publishes a list containing values of PNEC-environment (PNEC-ENV) and PNEC-minimum inhibitory concentration (PNEC-MIC) (34, 140). The PNEC-ENV values are intended to be protective of ecosystems, based on ecotoxicology data shared by AMR Industry Alliance members and a literature review (141, 142). The PNEC-MIC values are intended to be protective of resistance promotion and are based on the approach described in Bengtsson-Palme and Larsson (143). The values of both types of PNECs for the selected 40 antibiotics used in the present study are listed in Table 1. Note that the AMR Industry Alliance suggests that a substance-specific PNEC-ENV, PNEC-MIC, or the lowest of both values should be used in exposure assessments. We followed the latter recommendation in the present study.

#### Environmental risk assessment

In this study, the ratio of PEC to PNEC was used to calculate a risk quotient as an indicator of environmental exposure to individual chemical substances in the global river network. That is, the risk quotient for an individual substance *i* (RQ*_i_*; dimensionless) was calculated for every river reach:


(1) $$RQ_{i}=\frac{\mathrm{PE}C_{i}}{\mathrm{PNE}C_{i}}$$

where PEC*_i_* (ng L^−1^) was the predicted environmental concentration for substance *i* and PNEC for substance *i* (PNEC*_i_*; ng L^−1^) was chosen as the lower of the PNEC-ENV and PNEC-MIC values for each substance *i* listed in Table 1. The risk quotient provides a measure of the potential risk by indicating whether the concentration of a substance exceeds a level that could cause adverse effects. While it does not directly incorporate the probability of occurrence, it is a widely accepted method for screening-level assessments to prioritize areas or substances that may pose significant environmental risks (144–146).

In addition to the substance-specific risk quotients, a total risk quotient, RQ_tot_ (dimensionless), was calculated by summing the RQ*_i_* of each individual substance. This method was adapted by Zhan and Zhang (75) from the concentration addition concept (147) and was recently used to estimate overall risk of pesticides (76) and antibiotics (77) at the global scale:


(2) $$RQ_{\mathrm{tot}}=\;\underset{i=1}{\sum }\;RQ_{i}$$

This conservative approach ensures that locations with multiple antibiotics present are appropriately flagged for further investigation. However, it can lead to assumptions regarding AMR selection mechanisms that might not fully capture the complexity of these interactions. In addition, relying solely on cumulative risk could lead to inflated risk estimates if many substances are present at low concentrations. Thus, the maximum risk quotient (RQ_max_) of all substances was calculated for every river reach to provide a more comprehensive picture:


(3) $$RQ_{\max}=\max RQ_{i}$$

#### Defined daily doses

In terms of potential human exposure, and as described further below, the quantities of antibiotics in waters were expressed relative to their DDDs(g). According to the WHO, DDD is the “assumed average maintenance dose per day for a drug used for its main indication in adults” and was compiled for each antibiotic using the ATC/DDD index for 2022 (35). This index is based on the Anatomical Therapeutic Chemical (ATC) classification system developed by the WHO Collaborating Centre for Drug Statistics Methodology. If more than one DDD was assigned for an ATC depending on the route of administration, the value for oral route was selected. The DDD has been used as a surrogate for estimating potency of a substance when other ecotoxicological thresholds were not available (148).

#### Accepted daily intake

To further assess human health exposure, we compared our results with the ADI (µg kg^−1^ day^−1^) of the different antibiotics (74), defined as “an estimate amount of a substance in food or drinking water that can be consumed daily over a lifetime without presenting an appreciable risk to health” (149). ADI values were only available for 30 of the 40 antibiotics selected for inclusion in this study, as compiled by Khan and Nicell (2).

#### Human health risk assessment

No explicit benchmarks are currently available to quantify the potential effects of antibiotic residues in surface waters on human health. Therefore, for the purposes of the present study, a choice was made to calculate an aggregated indicator as a proxy for the exposure to all 40 selected antibiotics, by converting the instream concentration with respect to the substance's DDD and then summing these substance-specific dose concentrations to produce a net indicator of human exposure. This indicator is termed here as the “equivalent dose concentration” (*D*_eq_). First, the *D*_eq_ is calculated for each substance *i* (*D*_eq*,i*_) for each river reach, using the equation:


(4) $$D_{\mathrm{eq},i}=\frac{\mathrm{PE}C_{i}}{\mathrm{DD}D_{i}}$$

where *D*_eq*,i*_ is expressed in dose equivalents per liter (d_eq_ L^−1^) and DDD*_i_* is the DDD (g) of substance *i* for an adult based on the ATC/DDD index (35). Dose equivalent is defined here as the proportion of the DDD in the river reach (ng g^−1^). In analogy to the concentration addition concept (see above), the total equivalent dose concentration (*D*_eq,tot_) expressed in dose equivalents per liter (d_eq_ L^−1^) is then calculated as:


(5) $$D_{\mathrm{eq},\mathrm{tot}}=\;\underset{i=1}{\sum }\;D_{\mathrm{eq},i}$$

By assuming that a person consumes drinking water directly taken from surface water without further treatment, a total predicted daily intake of antibiotics (PDI_eq,tot_; µd_eq_ kg^−1^ day^−1^) is estimated using the following equation:


(6) $$\mathrm{PD}I_{\mathrm{eq},\mathrm{tot}}=\frac{D_{\mathrm{eq},\mathrm{tot}}\times \mathrm{DWI}\;}{\mathrm{BW}}$$

where DWI is the average drinking water intake (assumed to be 2 L day^−1^) and BW is an average body weight (assumed to be 60 kg). If this person consumes what we consider in this study as a proxy for high levels of equivalent dose concentrations (99th percentile; 29 µd_eq_ L^−1^), the total predicted daily intake of antibiotics would amount to 0.97 µd_eq_ kg^−1^ day^−1^. To compare this value with substance-specific ADIs, an equivalent predicted daily intake (PDI_eq*,i*_; µg kg^−1^ day^−1^) for each substance *i* was estimated using the following equation:


(7) $$\mathrm{PD}I_{\mathrm{eq},i}=\mathrm{PD}I_{\mathrm{eq},\mathrm{tot}}\;\times \mathrm{DD}D_{i}$$

The PDI_eq*,i*_ was then compared to the ADI of 30 substances with available data. In cases where the estimated daily intake exceeds the respective ADI, there is a potential risk to human health, which was the case for 16 out of the 30 substances. Therefore, we consider the 99th percentile of highest equivalent dose concentrations to be a meaningful first-order proxy to highlight river reaches that pose a potential health risk.

Following Richter et al. (150), people living within 10 km of a river are assumed to be potentially dependent on river services, such as water provision or groundwater recharge, making water quality an important factor of human health risk. Subsequently, the number of potentially affected people along rivers exposed to high levels of equivalent dose concentrations (99th percentiles) was assessed by using the population count inside associated reach catchments (average area of 15.7 km^2^), derived from the Gridded Population of the World dataset version 4 (151) as provided in RiverATLAS (129).

This approach has several uncertainties due to data limitations at a global scale, including: (i) the assumption that drinking water is taken from surface water without any further treatment, which is not the reality in many countries; (ii) the proxy “equivalent dose concentration” does not explicitly indicate risk; and (iii) the assumption that people living within 10 km of a river at risk are potentially exposed to the pollution is simplistic and does not account for several factors including variations in terrain, groundwater conditions, and water supply systems. Despite these uncertainties, it is important to note that conventional drinking water systems are not designed to remove antibiotics and are generally of limited effectiveness in doing so (152, 153). Countries with large populations situated near rivers with high concentrations of antibiotics may be at significant risk if they lack effective treatment systems. Importantly, our results are intended to serve as a preliminary guide for further research and should not be interpreted as a locally precise predictor of risk.

## Supplementary Material

pgaf096_Supplementary_Data

## Data Availability

The modeled results for every river reach in the global river network—that is, the PECs, the risk quotients, and the equivalent dose concentrations, can be accessed at https://doi.org/10.6084/m9.figshare.25829464 under a CC-BY-4.0 License. The source code is also available at the same link under a GNU General Public License v3.0. For additional information and updates on the HydroFATE model, visit https://www.hydrosheds.org/applications/hydrofate.
