# Correction to: Antibiotics in the global river system arising from human consumption

**DOI:** 10.1093/pnasnexus/pgaf181

**Published:** 2025-06-10

This is a correction to: Heloisa Ehalt Macedo, Bernhard Lehner, Jim A Nicell, Usman Khan, Eili Y Klein, Antibiotics in the global river system arising from human consumption, *PNAS Nexus*, Volume 4, Issue 4, April 2025, pgaf096, https://doi.org/10.1093/pnasnexus/pgaf096

In the originally published version of this manuscript, four numerical values reported in the abstract did not precisely match those presented in the final analysis. These values reflected an earlier version of the authors’ model output and, while not substantially incorrect, were not the final numbers. The abstract has been corrected as follows:

Original: “The presence of antibiotics in surface waters poses risks to aquatic ecosystems and human health due to their toxicity and influence on antimicrobial resistance. After human consumption and partial metabolism, antibiotic residues are excreted and undergo complex accumulation and decay processes along their pathway from wastewater to natural river systems. Here, we use a global contaminant fate model to estimate that of the annual human consumption of the 40 most used antibiotics (29,200 tonnes), 8,500 tonnes (29%) are released into the river system and 3,300 tonnes (11%) reach the world's oceans or inland sinks. Even when only domestic sources are considered (i.e. not including veterinary or industrial sources), we estimate that 6 million km of rivers worldwide are subject to total antibiotic concentrations in excess of thresholds that are protective of ecosystems and resistance promotion during low streamflow conditions, with the dominant contributors being amoxicillin, ceftriaxone, and cefixime. Therefore, it is of concern that human consumption alone represents a significant risk for rivers across all continents, with the largest extents found in Southeast Asia. Global antibiotic consumption has grown rapidly over the last 15 years and continues to increase, particularly in low- and middle-income countries, requiring new strategies to safeguard water quality and protect human and ecosystem health.”

Corrected: “The presence of antibiotics in surface waters poses risks to aquatic ecosystems and human health due to their toxicity and influence on antimicrobial resistance. After human consumption and partial metabolism, antibiotic residues are excreted and undergo complex accumulation and decay processes along their pathway from wastewater to natural river systems. Here, we use a global contaminant fate model to estimate that of the annual human consumption of the 40 most used antibiotics (30,300 tonnes), 9,500 tonnes (31%) are released into the river system and 3,250 tonnes (11%) reach the world's oceans or inland sinks. Even when only domestic sources are considered (i.e. not including veterinary or industrial sources), we estimate that 6 million km of rivers worldwide are subject to total antibiotic concentrations in excess of thresholds that are protective of ecosystems and resistance promotion during low streamflow conditions, with the dominant contributors being amoxicillin, ceftriaxone, and cefixime. Therefore, it is of concern that human consumption alone represents a significant risk for rivers across all continents, with the largest extents found in Southeast Asia. Global antibiotic consumption has grown rapidly over the last 15 years and continues to increase, particularly in low- and middle-income countries, requiring new strategies to safeguard water quality and protect human and ecosystem health.”

The authors would like to clarify that this discrepancy does not affect any of the conclusions or recommendations made in the paper. All figures and tables contain the correct values.

