# Supplementary material for: Antibiotics in the global river system arising from human consumption
Source: PNAS Nexus. 2025 Apr 22;4(4):pgaf096. doi: 10.1093/pnasnexus/pgaf096 (PMC12012769; doi:10.1093/pnasnexus/pgaf096)
Supplement: pgaf096_Supplementary_Data [file pgaf096_supplementary_data.pdf]

Supporting Information for

Antibiotics in the Global River System Arising from Human Consumption

Heloisa Ehalt Macedo[a*], Bernhard Lehner[a], Jim A. Nicell[b], Usman Khan[b], Eili Y. Klein[c,d]

[a]Department of Geography, McGill University, Montreal, QC, Canada H3A 0B9
[b]Department of Civil Engineering, McGill University, Montreal, QC, Canada H3A 0C3
[c]One Health Trust, Washington, DC, USA 20015
[d]Department of Emergency Medicine, John Hopkins School of Medicine, Baltimore, USA 21287

*Corresponding author
Email: heloisa.ehaltmacedo@mail.mcgill.ca

This PDF file includes:

Supporting text
Figures S1 to S3
Tables S1 to S4
SI References

Supporting Information Text

S1. Performance evaluation

The performance of the model was evaluated by comparing reported measured environmental concentrations (MECs) to predicted environmental concentrations (PECs) that had been estimated using HydroFATE for average-flow conditions at the coinciding river reaches. The goodness-of-fit indicators used to quantify model performance included the normalized root mean square error (NRMSE), the percentage of bias (PBIAS), and the coefficient of determination (R^2). HydroFATE's capability to predict risk exceedance adequately was also evaluated (see 'Risk assessment' section on how risk was assessed). To evaluate cases where MECs were classified as 'not detected' or 'not quantified', PECs at the same rivers locations were verified to determine if they were correctly predicted to be below either the limit of detection (LOD) or limit of quantification (LOQ), depending on what limits were reported in the respective studies. Furthermore, the sensitivity of model output to parameter and configuration settings was evaluated using scenario analyses to provide deeper insights into the capabilities and limitations of the model and test the model beyond average conditions.

Measured environmental concentrations of antibiotics in rivers. To evaluate the accuracy of PECs simulated by HydroFATE, MECs of 21 different antibiotics were gathered through an extensive literature search. In total, 5,793 MECs from 877 locations were compiled as reported in 65 publications. From these, 1,616 were above the limit of quantification (LOQ); i.e., concentrations that were above the minimum value that can be detected based on the analytical method used for quantifying contaminant concentrations in water samples. The MECs were collected as two separate sets (no overlap) in order to preserve their distinct characteristics that can be used for different evaluation purposes. Set 1 contains MECs of 21 antibiotics from 64 different studies, including amoxicillin, which is the antibiotic that is consumed the most worldwide; and Set 2 contains MECs of only 7 antibiotics, but from a single study[1]. The consistency of methodology and comprehensive distribution of the measurements worldwide make Set 2 a unique repository for a global evaluation.
Set 1 contains 2,538 MECs (including 1,066 above the LOQ) from 410 locations compiled from 64 different studies (for reference sources see Table S1). Based on MECs above LOQ, the average detected value is 261 ng L^{-1}, the median is 16 ng L^{-1}, the maximum is 65,000 ng L^{-1}, and the minimum is 0.015 ng L^{-1}. In order to be selected for inclusion as a MEC in the model evaluation, the literature source must have reported the specific location (i.e., in the form of coordinates, river names, or river intersections) of the measurements. In addition, we discarded any MEC where the literature source explicitly mentioned that the dominant use of antibiotics in the catchment feeding the river was associated with veterinary or industrial activities. The last was an effort to exclude known cases where measurements and predictions are by default expected to be incomparable since the current version of HydroFATE is not adapted to account for these sources. While databases such as NORMAN[2] and PHARMS-UBA[3] were considered and used as a guide, we collected the MECs from literature ourselves to ensure the quality and accuracy of the data. This approach allowed us to verify that the values were not spatial averages, confirm their exact location (as existing databases do not always include geographic coordinates), and ensure the MECs fulfilled our requirements.

Set 2 contains 3,255 MECs (including 550 above the LOQ) from 467 locations from the study of Wilkinson, et al. [1]. Their study reported on the monitoring of 61 different pharmaceuticals in 1,052 sampling sites in 104 countries, including 13 antibiotics. From these, 7 antibiotics matched our selection of 21 evaluation substances: ciprofloxacin, clarithromycin, erythromycin, lincomycin, sulfamethoxazole, tetracycline, and trimethoprim. Not all sampling sites could be used because some locations could not be clearly associated with the river network. Based on MECs above LOQ, the average detected value is 256 ng L^{-1}, the median is 44 ng L^{-1}, the maximum is 15,000 ng L^{-1}, and the minimum is 1.2 ng L^{-1}.

As a general shortcoming, reports of most recorded MECs did not include comprehensive information on the specific river characteristics, such as width or average discharge, which would

have helped in identifying the precise location in the river network, nor on the conditions under which the measurement was taken, such as actual discharge amount or flow season (low, average, or high flow). Therefore, the location and value of the reported MECs may not always accurately correspond to the georeferenced river network location and/or the assumed flow conditions in HydroFATE.

Comparison of measured and predicted environmental concentrations. Evaluations were performed separately for each of the two sets of MECs to compare them with predicted environmental concentrations (PECs) at average-flow conditions. Results revealed that model predictions were generally within a reasonable range of concentrations reported in the literature, with 552 (51%) and 371 (67%) of the predicted values being within one order of magnitude of the measurements for Set 1 and Set 2, respectively (see Table S2), which is generally considered adequate for these types of screening models[68,69]. In terms of goodness-of-fit indices, PBIAS indicates that PECs were generally lower than MECs for Set 1, but generally higher than MECs in Set 2. The contrasting biases from both datasets combined to an overall low positive PBIAS when both sets were merged. The R^2 was low for both sets (0.04 and 0.18 for Set 1 and Set 2, respectively); however, this index may not be a good indicator of performance for data that have high uncertainties for both predictions and measurements.

The regional distributions of the measurements shown in Figure S1 (i.e., panel (a) for Set 1 and panel (b) for Set 2) reveal that most predictions were higher than measurements for the African continent, whereas in Asia, Europe, and Central America, the predictions were generally lower than the measurements. Predictions using extrapolated values of consumption (triangular dots in Figure S1(a)-(d)), were generally higher than measurements for Set 1, while the same trend was not observed for Set 2.

In terms of model performance with respect to predictions for specific substances, model estimates for sulfamethoxazole (sulfonamides) and trimethoprim produced the best agreement with measurements for both Sets 1 and 2. This agreement can be seen in panels (c) and (d) of Figure S1 and in the combination of goodness-of-fit indices in Table S2 (i.e., higher R^2 and number of PECs within one order of magnitude from MECs; lower NRMSE and PBIAS).

Panels (e) and (f) of Figure S1 show how the model behaved in relation to river sizes (i.e., based on average river discharge) and level of urbanization (i.e., based on upstream urban extents). In general, the model estimates tended to be lower than the measurements in rural areas and in larger rivers, and higher downstream of urban areas and in smaller streams. As model results and measurements can be expected to be particularly error prone in small urban streams, where highly localized contamination occurs, this confirms a general model trend to estimate PECs below MECs in presumably more reliable situations.

Regarding the ability of the model to predict concentrations above or below risk levels, only 4% of MECs above LOQ from both sets were predicted erroneously as being above a protective threshold (PNEC, see details below). That is, the model rarely overpredicted risk. Corroborating this tendency of underpredicting risk, the model only was able to confirm concentrations above the protective threshold for 35% of MECs that reported an exceedance of PNEC. While this underprediction could be attributed to veterinary or industrial sources affecting the MECs, no conclusive statements are currently possible due to high uncertainties also involving other aspects of the analysis, including model parameters such as wastewater removal and instream decay constants.

From the 4,177 MECs below the LOQ, the associated studies reported the LOQ detection threshold for 3,806. For these, HydroFATE successfully predicted 70% of PECs to be below the respective LOQ threshold. Inversely, HydroFATE predicted risk incorrectly for only 5% of MECs below the LOQ.

Model sensitivity to different scenarios. Model simulations were conducted to analyze the sensitivity of model results to uncertainties associated with each investigated parameter and configuration setting. Table S3 shows the settings for 2 main scenarios and 16 sub-scenarios. Scenario 1 represents baseline conditions using input parameters and model configurations that

are expected to yield the most plausible predictions for average-flow conditions based on reported values in literature and in Grill, et al. [70] for the direct discharge coefficients. Scenarios 2 and 3 were implemented to create error bars around the results of baseline Scenario 1; i.e., in each iteration the baseline configuration was maintained but one target parameter or configuration setting was changed towards their limit that induces lower or higher contamination, respectively. This included the implementation of low-flow conditions instead of average discharge in Scenario 3. Scenario 4 represents an overall worst-case scenario. Thus, all parameter and configuration settings were chosen to predict the highest possible antibiotic concentrations in the river network; i.e., Scenario 4 assumes that there is no removal due to metabolism (which would be the case for a substance that is flushed down the drain rather than ingested), nor treatment or natural attenuation, and no decay in rivers and lakes (i.e., reflecting a compound that is persistent in the environment).

In all scenarios, 'most plausible' parameter values were taken from Table 2 in the main text and scenario permutations for the parameters were then executed within ranges as specified in Table S3. The excretion fraction was investigated for a range of ±10% around the 'most plausible' value for each substance. This range was chosen since most compiled excretion fraction values by Khan[72] vary by plus or minus 10%, from which the average for the baseline scenario was derived. Removal efficiencies in wastewater treatment facilities were allowed to range from 0% to 100%, for all levels of treatment. Note that, in reality, certain substances can even exceed this range. For example, based on a literature review of 36 publications with 190 removal efficiencies for sulfamethoxazole, Straub[73] reported a range extending from -1100% (i.e., a higher concentration in the effluent than in the influent due to the transformation of metabolization products back to the parent compound, sulfamethoxazole, in WWTPs) to a maximum of 100%. The urban and rural direct discharge coefficients (*ddc*) apply in situations where no wastewater treatment is involved and represent a proxy parameter that determines the proportion of the contaminant load that remains after processes of natural attenuation, such as absorption in soils or deposition in land surface depressions. For baseline conditions, Scenario 1 follows Grill, et al.[70] and Ehalt Macedo, et al.[71], using 0.8 as the urban *ddc* and 0.5 as the rural. The higher factor for urban areas is due to the presence of impervious surfaces leading to more direct disposal of wastewater to nearby rivers and streams. These values heavily rely on assumptions since the processes involved are complex and not well understood, and little evidence exists in literature to verify their plausibility. For this reason, the *ddc* parameters were investigated at their full range of possibilities: i.e., *ddc* values were allowed to range from 0 to 1 in Scenarios 2 and 3. The instream decay constants were investigated from 0 to 5.36 day^{-1}, representing conditions from no decay to the maximum value of any substance listed in Table 2 in the main text. Instream decay is expected to have a larger impact in river reaches (or at MEC locations, in this analysis) where the travel time of the contaminant load is longer (i.e., main sources of contaminants are far from the measurement locations), which grants more time for decay processes to remove the contaminant.

The model configuration of 'lake removal' was investigated as ranging from 'full removal' to 'no removal' in Scenarios 2 and 3. In Scenario 1, the applied mixing process within lakes (i.e., using the 'completely stirred reactor' (CSTR) method) produces results for each lake that fall between these range limits. The choice of river discharge levels affects the PEC calculation in every river reach. Within the different scenarios, possible discharge estimates represent either average conditions (i.e., long-term annual average from 1971-2000), the same as Scenario 1, or low-flow conditions (i.e., discharge value of lowest month within an average year). Finally, the hypothesis that countries at the same economic level consume the same per-capita amount of a substance was tested by assuming that antibiotics are only used in countries that report on them.

Figure S2 presents the findings of the sensitivity assessment with regards to parameter settings. Panel (a) displays sensitivity related to variations in the excretion fractions. In HydroFATE, calculated PECs are directly proportional to the excretion fraction, and since the range reported by the literature is small, the error bars of PECs are also small. Panels (b), (c), and (d) show the influence of varying removal levels (i.e., wastewater treatment removal efficiencies or direct discharge coefficients) associated with different types of contaminant pathways (i.e., treated versus untreated urban or rural). Panel (e) illustrates the sensitivity related to variations in the instream decay constants. The large ranges are due to the large uncertainties reported in

literature, involving the different processes of instream decay which are highly variable and depend on various physical, chemical, and biological parameters of the local environment.

Regarding model configuration settings, panel (a) of Figure S3 shows the model's sensitivity towards variations in lake removal methods. The CSTR method delivers average results, a compromise between likely overestimation of concentrations (no contaminant removal in lakes) and underestimation of concentrations (full removal in lakes). Panel (b) shows the sensitivity of results caused by presumed river discharge conditions. For some locations, the difference between MECs and PECs would be substantially decreased if using low-flow discharge conditions (instead of average flow conditions) in the model simulations. This analysis demonstrates that cases in which PECs were too low could, in part, be explained by uncertainties within the measurements (i.e., measurements potentially taken at low-flow conditions rather than errors in the model predictions). Panel (c) shows the influence of extrapolating the per-capita antibiotics consumption for countries that do not report any data on model predictions. If the extrapolation method was not applied, i.e., no antibiotic consumption was assumed for countries that were not reporting (as represented by Scenario 2h), for 179 MECs above the LOQ the corresponding PECs would be simulated as zero. Most of these MECs were located in Africa, as shown in panels (a) and (b) of Figure S1, and therefore the omission of countries that did not report antibiotics consumption data could lead to a considerable under-assessment of risk in this continent.

Finally, panel (d) of Figure S3 illustrates the worst-case Scenario 4 which results in the maximum values of PECs based on parameter and configuration settings that correspond to the elimination of all contaminant removal processes. Most upper limits of the error bars are above the 1:1 line, indicating the critical importance of the removal simulations in the model.

Discussion on model performance. The main goal of the model evaluation was to investigate the uncertainties of HydroFATE and show whether a model that is global and based on readily available datasets can deliver robust results that are adequate for screening purposes and for subsequent risk assessments. As such, processes simulated in the HydroFATE model are subject to many uncertainties, including: (1) the separation of contaminant pathways between those that are treated and untreated is based on the distribution of WWTPs, population density and sanitation statistics, relying on global datasets that are subject to high uncertainties; (2) wastewater treatment removal processes are simplified and represented by removal efficiencies, only distinguished between primary, secondary, and advanced levels of treatment, and ignoring differences between specific treatment types and local circumstances; (3) natural attenuation processes are simplified using two coefficients (i.e., rural *ddc* and urban *ddc*) for which estimated values mostly rely on assumptions in the absence of measurements; (4) instream decay processes (i.e., deposition, adsorption, photodegradation, and bioaccumulation) are simplified by using a single first-order decay function; (5) lake removal processes are simplified and also reliant on the instream decay constant; and (6) HydroFATE is a global-scale model based on a steady-state approach using long-term average or minimum discharge, which fails to capture the hydrologic seasonality or any temporal flow dynamics.

These uncertainties affect the predictions, depending on the model's sensitivity to parameters and model settings. Our sensitivity analysis revealed that the model sensitivity depends on the characteristics, locations, and reliability of MECs in the river network. For example, we found that predictions are highly sensitive to the uncertainties related to removal and attenuation processes within the pathway from the sources of antibiotics to the river; i.e., removal by wastewater treatment or natural attenuation (*ddc*). During the evaluation of results, MECs were separated in two sets with distinct characteristics. Overall, the model performance was similar for both sets (Table S2), reinforcing the model's robustness when tested against validation data with different specifications. In comparison with studies using similar water quality models at large spatial scales[68,74,75], our study incorporates a wider range of substances, detailed spatial resolution and better representation of contaminant pathways, providing a more comprehensive assessment of antibiotic exposure and risks in the global river system. For example, while Oldenkamp et al[68] reported that 95% of their results were within an order of magnitude, our study achieved only 67%. However, it is important to note the contextual differences between these studies.

Oldenkamp et al[68] validation focused on two specific basins in Europe (Rhine and Ouse), which benefit from highly reliable input data and established monitoring systems. Furthermore, their model resolution was slightly lower (1-km), which tends to moderate predictions and reduce variability. In contrast, our study is the first of its kind to assess antibiotic pollution at a global scale with a much higher spatial resolution (500-m). This inherently increases the complexity and uncertainty of the model due to the variability in river characteristics, population density, sanitation practices, and other environmental factors across different regions. Given these differences, we believe a direct comparison may not be entirely fair or reflective of the challenges involved in global-scale modeling.

To gain additional insights into the model's performance, several outliers (referred to by corresponding identification (ID) numbers of the ellipses shown in Figure S1) were analyzed through comprehensive examination of original studies, satellite imagery, and additional geospatial information. We found that outlier predictions that were found to be too high in comparison to measurements may be due to a variety of factors, as follows: extrapolated national consumption rates, which can be lower (or higher) in reality (ID 1); concentrations measured in periods of high streamflow (ID 2); and WWTP outfall locations potentially being erroneously placed in the wrong river, especially for small streams in urban areas (ID 5). In contrast, predictions that were simulated to be too low in comparison to measurements may be due to: concentrations measured in periods of low flow (ID 3); the possibility of contributions from other sources of antibiotics, such as veterinary or industrial (IDs 4 and 7); effluents from WWTPs with simulated outfall locations in the ocean which in reality might discharge into coastal rivers (ID 6); and concentrations measured in rivers immediately downstream of WWTPs before the contaminant load fully mixed with the waterbody or downstream of other point sources (e.g., industry, hospitals) not accounted for in the model (IDs 8 and 9). While these are plausible reasons for discrepancies between measured and predicted concentrations, it should be noted that even predictions that were in good agreement with measurements might be affected by one or more of these uncertainties. The outlier analysis therefore indicates that MECs also present their own uncertainties and could incorrectly imply poor model performance. Despite our best efforts, we cannot guarantee that all selected MECs do not include veterinary or industrial sources. Regardless, the removal of all outliers did not greatly improve the goodness-of-fit indices for model evaluation, in part corroborating the limited ability of statistical indicators to holistically assess model performance in cases of high uncertainties in both predictions and measurements[74,76].

Despite the model shortcomings and uncertainties summarized above, the best agreement between predictions and measurements were observed for substances with the largest number of measurements and/or detections, as shown in panels (c) and (d) of Figure S1 and Table S2. This lends a higher degree of confidence in the model when it is used to simulate prevalent and common conditions than when modelling special situations such as those exemplified by the outliers. From this, it can be concluded that the model performs adequately for average conditions but may not be relied upon to accurately simulate special or local conditions, not least due to the characteristics of the data inputs (i.e., long-term averages).

Table S1. Literature sources and numbers of measured environmental concentrations (MECs), total and above the Limit of Detection (LOD). The “Substance” index values refer to Table 2 in the main text.

Reference	Country	Substance																					Number of MECs	Number of MECs above LOD
		1	2	3	4	5	8	9	11	12	15	17	18	19	21	25	27	28	29	30	32	33		
Adachi, et al. [4]	Japan			X												X				X			17	17
Agunbiade and Moodley [5]	South Africa			X		X																	6	6
Arikan, et al. [6]	United States		X									X						X					7	0
Arsand, et al. [7]	Brazil		X	X	X			X					X	X		X							14	14
Aydin and Talinli [8]	Turkey	X	X	X							X												16	16
Bagnis, et al. [9]	Kenya		X	X						X	X	X	X									X	70	30
Barber, et al. [10]	United States	X	X	X		X		X			X	X	X	X	X	X		X		X		X	52	7
Batt, et al. [11]	United States		X	X								X	X	X									20	4
Bendz, et al. [12]	Sweden		X										X										4	4
Böger, et al. [13]	Brazil	X	X	X				X								X		X					12	9
Calamari, et al. [14]	Italy	X		X			X			X	X				X		X					X	49	24
Camacho-Muñoz, et al. [15]	Spain		X										X										8	1
Carlson, et al. [16]	Canada		X	X						X	X		X				X						30	10
Chang, et al. [17]	China		X	X							X	X				X				X	X	X	34	16
Chau, et al. [18]	Vietnam		X			X		X		X	X		X				X		X			X	261	8
Chaves, et al. [19]	Brazil		X																				3	2
Chitescu, et al. [20]	Romania		X	X							X		X			X		X	X				104	23
Choi, et al. [21]	South Korea			X									X										8	8
Deng, et al. [22]	Hong Kong			X																			7	0
Dinh, et al. [23]	France	X	X	X							X	X	X		X	X				X			36	12
Ekberg and Pletsch [24]	United States		X										X										34	29
Feitosa-Felizzola and Chiron [25]	France		X	X				X		X													4	0
Fernandes, et al. [26]	Portugal		X	X				X		X			X			X				X			28	5
Fick, et al. [27]	Sweden		X	X				X		X	X	X	X	X		X							50	17
Fick, et al. [28]	India			X									X			X				X			4	4
Finnegan, et al. [29]	United States	X	X	X		X		X			X	X	X		X	X		X		X			52	17
Fonseca, et al. [30]	Spain		X	X				X		X	X	X	X	X		X							100	18
Hanna, et al. [31]	India		X	X			X									X				X			30	18
Joshua, et al. [32]	India		X				X						X						X				44	43
K'Oreje, et al. [33]	Kenya		X										X										14	11
Kairigo, et al. [34]	Kenya		X	X									X			X		X					6	1
Kandie, et al. [35]	Kenya		X										X										64	25
Kasprzyk-Hordern, et al. [36]	UK<br>Poland	X	X								X		X						X				30	9
Kasprzyk-Hordern, et al. [37]	UK	X	X								X		X						X				37	23
Khan, et al. [38]	Pakistan		X	X				X	X	X	X	X	X	X	X	X		X		X	X		163	111
Khan, et al. [39]	Sweden		X	X				X		X	X		X			X		X		X			9	7
Kim and Carlson [40]	United States		X								X	X						X					16	15
Kunkel and Radke [41]	Germany		X																				1	1
Li, et al. [42]	Sweden<br>Germany		X																				6	6
Locatelli, et al. [43]	Brazil	X	X	X	X	X		X				X	X			X							40	28
Loper, et al. [44]	United States		X	X				X			X		X							X			48	18
López-Serna, et al. [45]	Spain		X	X				X		X	X	X	X			X	X	X	X	X			252	120
Low, et al. [46]	Malaysia		X	X				X		X	X	X	X			X			X	X			20	3
Luo, et al. [47]	China		X	X							X	X	X							X			37	22
Managaki, et al. [48]	Vietnam<br>Japan		X					X		X	X		X								X		29	25
Osorio, et al. [49]	Spain		X	X				X		X	X	X	X				X	X	X	X	X		36	30
Paíga, et al. [50]	Portugal		X	X				X		X			X										25	7
Rivera-Jaimes, et al. [51]	Mexico		X										X										4	4
Sharma, et al. [52]	India		X	X																			26	17

Shimizu, et al. [53]	Vietnam Philippines		X		X	X	X	X	X			X				X	40	21
Sim, et al. [54]	South Korea		X	X			X		X							X	10	4
Sörengård, et al. [55]	Sweden		X			X	X	X	X	X			X				24	5
Spongberg, et al. [56]	Costa Rica		X	X		X	X	X	X	X	X	X		X		X	275	44
Stipaničev, et al. [57]	Macedonia		X	X	X		X		X				X				30	17
Tamtam, et al. [58]	France		X	X					X		X			X			20	12
ter Laak, et al. [59]	Netherlands		X			X	X		X	X					X		17	10
Thomas and Hilton [60]	UK		X				X		X								18	3
Tong, et al. [61]	China			X							X			X			6	6
Valdés, et al. [62]	Argentina			X		X											4	2
Vieno, et al. [63]	Finland			X							X			X			3	0
Vilimanovic, et al. [64]	United States		X			X	X		X								36	35
Wagil, et al. [65]	Poland			X							X						26	5
Wille, et al. [66]	Belgium		X						X								6	5
Zhang, et al. [67]	China	X	X	X			X				X	X		X		X	56	52
**Total**	**World**																**2 538**	**1 066**

Table S2. Evaluation of PECs estimated at average-flow conditions in relation to MECs above LOQ, separated for Sets 1 and 2, using goodness-of-fit indices and other statistics. R^2 is the coefficient of determination, NRMSE is the normalized root mean square error, PBIAS is the percent bias.

**Substance group** Substance	**Set**	**Number of MECs (in countries with extrapolated consumption)**	**R^2**	**NRMSE (%)**	**PBIAS (%)**	**Number of MECs in risk (% of total)**	**Correct risk/ no-risk detection (%)**	**PECs within one order of magnitude (% of total)**
**Penicillins**								
Amoxicillin	1	25 (0)	0.00	151	30	3 (12)	33/91	12 (48)
	2	–	–	–	–	–	–	–
Ampicillin	1	3 (0)	0.94	347	-99	3 (100)	0/ -	0 (0)
	2	–	–	–	–	–	–	–
**Sulfonamides**								
Sulfamethoxazole	1	227 (52)	0.64	62	-43	23 (10)	22/99	150 (66)
	2	190 (50)	0.37	107	54	26 (14)	52/92	147 (77)
**Fluoroquinolones**								
Ciprofloxacin	1	100 (14)	0.00	192	31	33 (33)	42/91	62 (62)
	2	71 (30)	0.01	4936	2099	28 (39)	36/60	30 (42)
Levofloxacin	1	4 (0)	0.58	125	162	0	– /100	2 (50)
	2	–	–	–	–	–	–	–
Ofloxacin	1	72 (1)	0.74	103	-96	7 (10)	0/100	9 (12)
	2	–	–	–	–	–	–	–
Norfloxacin	1	68 (0)	0.04	106	-86	7 (10)	0/100	22 (32)
	2	–	–	–	–	–	–	–
**Cephalosporins**								
Cefalexin	1	6 (0)	0.02	369	405	1 (17)	100/60	3 (50)
	2	–	–	–	–	–	–	–
Ceftriaxone	1	10 (0)	0.23	88	45	0 (0)	– /100	2 (20)
	2	–	–	–	–	–	–	–
Cefotaxime	1	3 (0)	0.83	87	-57	0 (0)	– /100	3 (100)
	2	–	–	–	–	–	–	–
**Lincosamides**								
Clindamycin	1	15 (0)	0.06	117	-89	1 (7)	0/100	11 (73)
	2	–	–	–	–	–	–	–
Lincomycin	1	39 (7)	0.00	851	382	0 (0)	– /92	20 (51)
	2	24 (2)	0.00	318	100	3 (12)	33/81	8 (34)
**Macrolides**								
Clarithromycin	1	73 (1)	0.18	98	-56	4 (5)	0/99	50 (68)
	2	48 (16)	0.09	149	-47	11 (23)	27/92	26 (54)
Erythromycin	1	89 (4)	0.01	102	-93	1 (1)	0/100	33 (37)
	2	25 (10)	0.72	58	-40	3 (12)	100/100	15 (60)
Azithromycin	1	32 (4)	0.00	107	-84	13 (41)	23/100	15 (47)
	2	–	–	–	–	–	–	–
Roxithromycin	1	21 (0)	0.01	109	-86	0 (0)	– /100	6 (29)
	2	–	–	–	–	–	–	–
Spiramycin	1	17 (2)	0.09	117	-97	0 (0)	– /100	5 (29)
	2	–	–	–	–	–	–	–
**Tetracyclines**								
Tetracycline	1	34 (0)	0.03	128	-98	0 (0)	– /100	3 (9)
	2	4 (0)	0.20	229	-99	0 (0)	– / 100	0 (0)
Doxycycline	1	46 (1)	0.00	101	-99	3 (6)	0/100	15 (33)
	2	–	–	–	–	–	–	–
**Trimethoprim**								
Trimethoprim	1	165 (35)	0.47	92	13	9 (5)	56/99	123 (75)
	2	188 (57)	0.42	168	142	9 (5)	78/91	145 (77)
**Amphenicols**								
Chloramphenicol	1	17 (3)	0.07	105	-69	0 (0)	– /100	6 (35)
	2	–	–	–	–	–	–	–
**Total**	1	1066 (124)	0.04	100	-50	108 (10)	27/98	552 (51)
	2	550 (165)	0.18	179	133	80 (15)	47/89	371 (67)
**Total (combined)**		**1616 (289)**	**0.04**	**110**	**11**	**190 (8)**	**35/96**	**923 (57)**

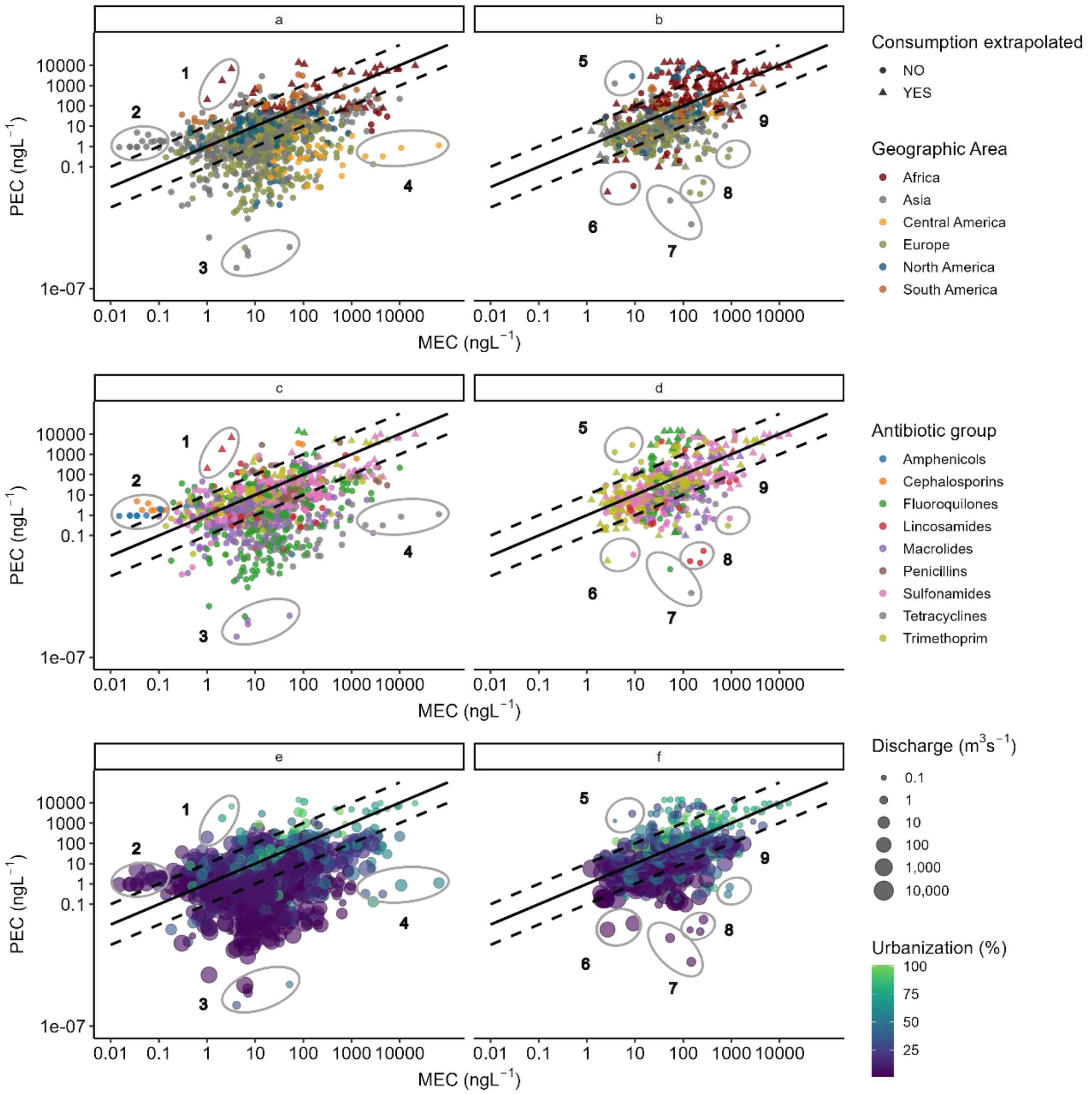

Fig. S1. Evaluation of the concentrations of 21 antibiotics in the global river system estimated using HydroFATE. Individual points represent comparisons between the measured (MEC) and the predicted (PEC) environmental concentrations at average-flow conditions in the same river reach. The left-hand panels (a), (c) and (e) represent the MEC/PEC pairs for Set 1 (see text for details); the right-hand panels (b), (d) and (f) represent the MEC/PEC pairs for Set 2. The colours in panels (a) and (b) represent the geographic regions in which the measurement is located. The colours in panels (c) and (d) represent the antibiotic group to which each of the pairs belongs. The point size in panels (e) and (f) represents the annual river discharge and the point colour represents the percent urban extent upstream of the river reach. The point shapes in panels (a) to (d) indicate if the PEC for that pair was estimated using extrapolated or reported values of country-level consumption. The black solid line in each panel represents the 1:1 correspondence line between predicted and measured concentrations and the black dashed lines represent the error lines corresponding to one order of magnitude. The numbered ellipses encircling 9 groups of outliers are further discussed in the text.

Table S3. Scenarios designed to represent plausible parameter and model configuration settings to simulate the global distribution of antibiotics in rivers. Parameters that are substance-specific were described in Table 2 in the main text. 'Urban ddc' and 'rural ddc' represent the direct discharge coefficient used in urban and rural areas respectively (values follow Grill, et al. [70] and Ehalt Macedo, et al. [71]). 'Lake removal' represents the method used to account for decay processes in lakes. 'Discharge condition' identifies if the predicted concentration was calculated using average or low-flow discharge. 'National extrapolation of consumption' identifies if the consumption was extrapolated for countries without reported data.

			Parameter settings					Configuration settings		
	Scenario	Parameter or configuration modified from baseline (only for sub-scenarios)	Excretion fraction	Wastewater treatment removal efficiency (%)	Direct discharge coefficient: Urban *ddc*	Direct discharge coefficient: Rural *ddc*	Instream decay constant (day^{-1})	Lake removal	Discharge condition	National extrapolation of consumption
**1**	***Baseline, average-flow***		TABLE 2	TABLE 2	0.8	0.5	TABLE 2	CSTR*	Average-flow	Yes
**2**	***Lower contamination limits***									
2a		*Excretion fraction*	– 0.1	TABLE 2	0.8	0.5	TABLE 2	CSTR	Average-flow	Yes
2b		*Wastewater treatment removal efficiency*	TABLE 2	100	0.8	0.5	TABLE 2	CSTR	Average-flow	Yes
2c		*Urban ddc*	TABLE 2	TABLE 2	0	0.5	TABLE 2	CSTR	Average-flow	Yes
2d		*Rural ddc*	TABLE 2	TABLE 2	0.8	0	TABLE 2	CSTR	Average-flow	Yes
2e		*Instream decay constant*	TABLE 2	TABLE 2	0.8	0.5	5.36	CSTR	Average-flow	Yes
2f		*Lake removal*	TABLE 2	TABLE 2	0.8	0.5	TABLE 2	Full removal	Average-flow	Yes
2g		*Discharge condition*	this scenario was not implemented due to being the same as Scenario 1							
2h		*Consumption extrapolation*	TABLE 2	TABLE 2	0.8	0.5	TABLE 2	CSTR	Average-flow	No
**3**	***Upper contamination limits***									
3a		*Excretion fraction*	+ 0.1	TABLE 2	0.8	0.5	TABLE 2	CSTR	Average-flow	Yes
3b		*Wastewater treatment removal efficiency*	TABLE 2	0	0.8	0.5	TABLE 2	CSTR	Average-flow	Yes
3c		*Urban ddc*	TABLE 2	TABLE 2	1	0.5	TABLE 2	CSTR	Average-flow	Yes
3d		*Rural ddc*	TABLE 2	TABLE 2	0.8	1	TABLE 2	CSTR	Average-flow	Yes
3e		*Instream decay constant*	TABLE 2	TABLE 2	0.8	0.5	0	CSTR	Average-flow	Yes
3f		*Lake removal*	TABLE 2	TABLE 2	0.8	0.5	TABLE 2	No removal	Average-flow	Yes
3g		*Discharge condition*	TABLE 2	TABLE 2	0.8	0.5	TABLE 2	CSTR	Low-flow	Yes
3h		*Consumption extrapolation*	this scenario was not implemented due to being the same as Scenario 1							
**4**	***No removal***		1	0	1	1	0	No removal	Low-flow	Yes

*CSTR: completely stirred reactor

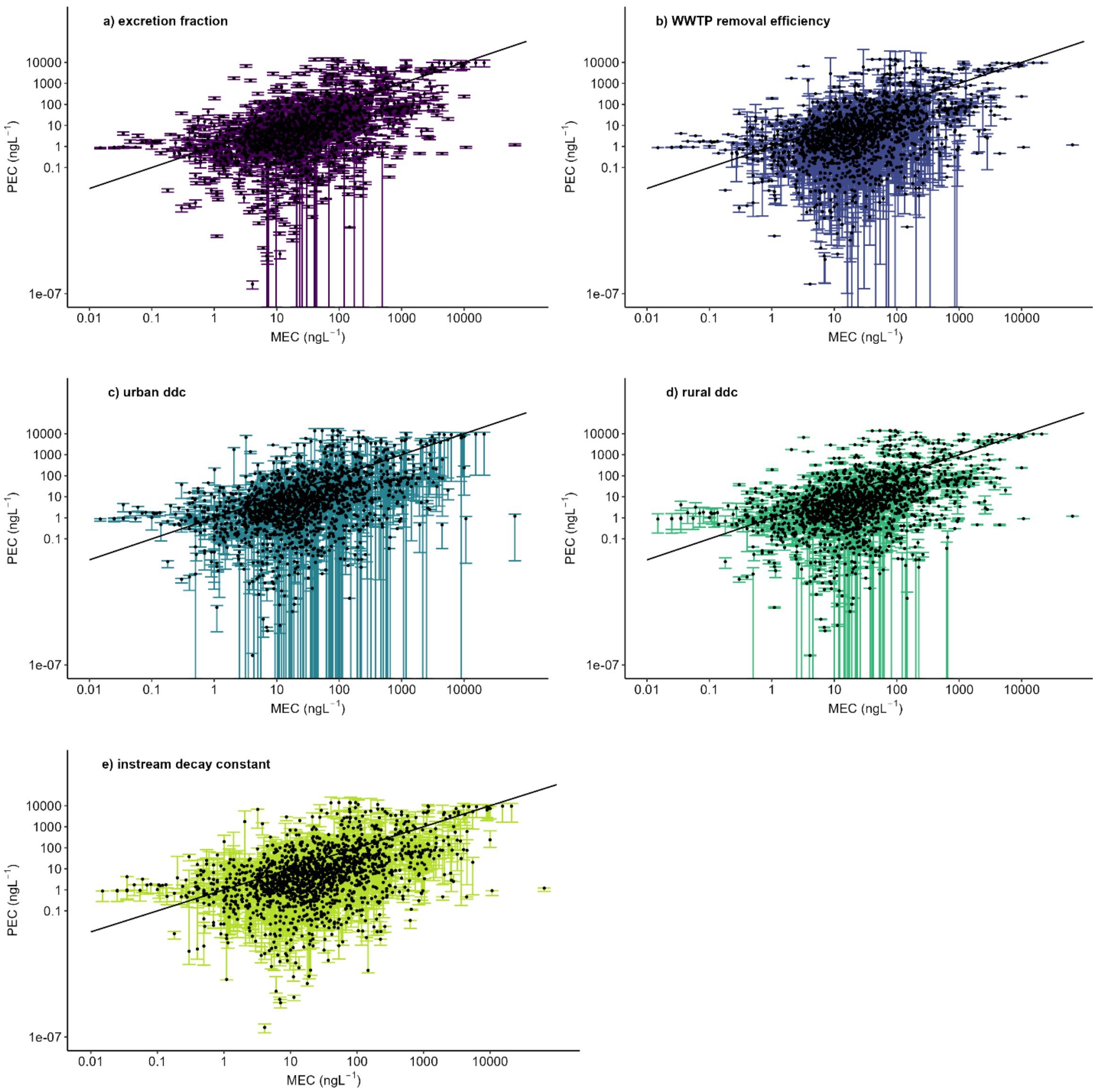

Fig. S2. Sensitivity of estimated antibiotic concentrations to changes in HydroFATE parameters. Black points represent comparisons between the measured (MEC) and the predicted (PEC) environmental concentrations for Scenario 1 (baseline). The black line in each panel represents the 1:1 correspondence line between predicted and measured concentrations. The error bars represent the range of the resulting PECs using the range of each input parameter as listed for the different scenarios in Table S3: (a) excretion fraction for Scenarios 2a and 3a; (b) wastewater treatment removal efficiency for Scenarios 2b and 3b; (c) urban ddc for Scenarios 2c and 3c; (d) rural ddc for Scenarios 2d and 3d; and (e) instream decay constant for Scenarios 2e and 3e. Error bars that extend below 10^{-7} ng L^{-1} may include predicted zero concentrations.

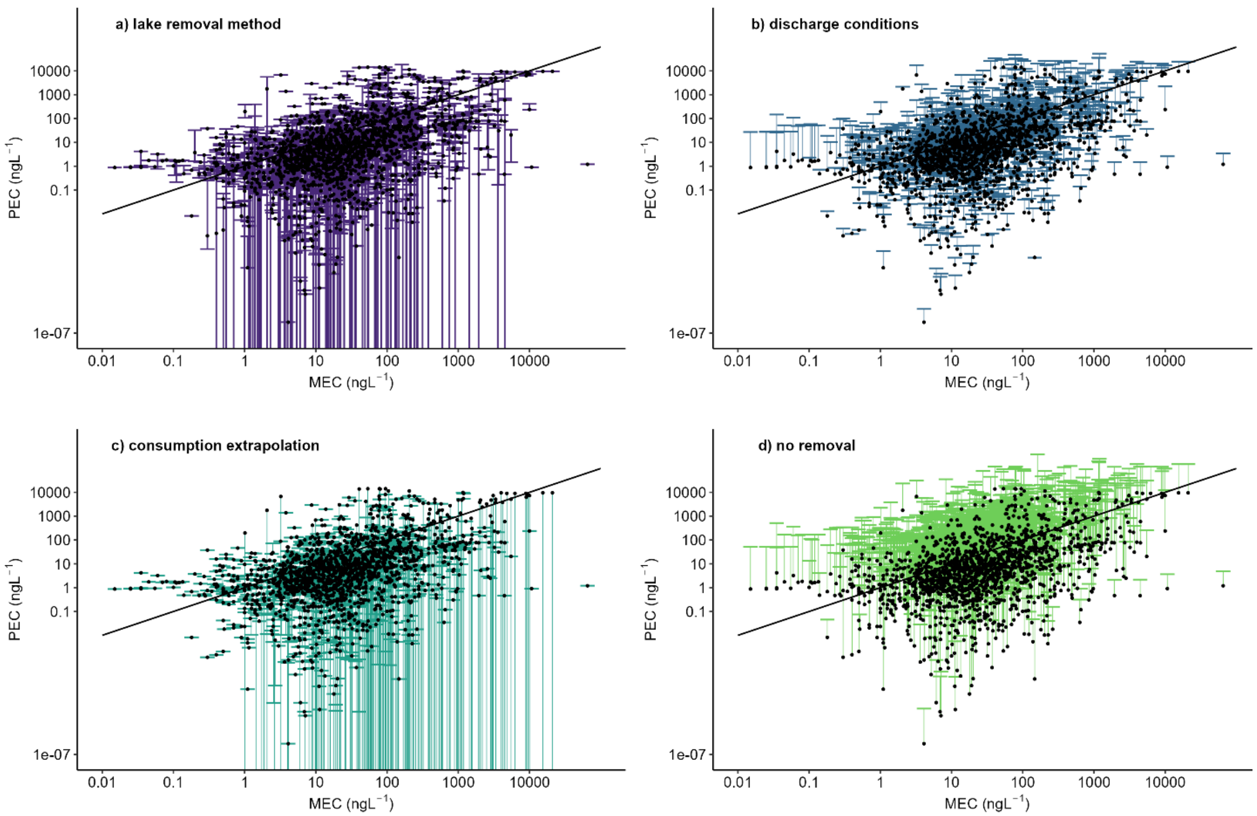

Fig. S3. Sensitivity of estimated antibiotic concentrations to changes in HydroFATE model configurations. Black points represent comparisons between the measured (MEC) and the predicted (PEC) environmental concentrations for Scenario 1 (baseline). The black line in each panel represents the 1:1 correspondence line. The error bars represent the range of the resulting PECs using the different methods to represent relevant processes as listed for the different scenarios in Table S3: (a) lake removal method for Scenario 2f and 3f; (b) discharge conditions for Scenario 3g; and (c) extrapolation of national per-capita consumption for Scenario 2h. Panel (d) represents a worst-case scenario using parameter and configuration settings as listed for Scenario 4 assuming no removal processes. Error bars that extend below 10^{-7} ng L $^{-1}$ may include predicted zero concentrations.

Table S4. Length of rivers presenting high environmental risk from antibiotic concentrations at low-flow conditions by country/territory. '$RQ_{tot} \geq 1$' accounts for all river reaches where the sum of individual risk quotients for all 40 substances equals or exceeds 1; '$RQ_{max} \geq 1$' accounts for all river reaches where the individual RQ of at least one substance equals or exceeds 1; '$D_{eq} \geq 99^{th}$ percentile' accounts for those river reaches that represent the 99th percentile with highest cumulative equivalent dose concentration(i.e., 29 μD_{eq} L^{-1}; see explanations in main text). '% of total' represents the percentage of length compared to all rivers in the national or global river network, respectively, exceeding a long-term average flow of 0.1 m^3 s^{-1}(23.8 million km).

Country/ Territory	***Environmental exposure***							***Human health exposure***		
	$RQ_{tot} \geq 1$		$RQ_{max} \geq 1$			$RQ_i \geq 1$ for at least 10 substances		$D_{eq} \geq 99^{th}$ percentile		
	Length of rivers (10^3 km)	% of total	Length of rivers (10^3 km)	% of total	Most contributing substance	Length of rivers (10^3 km)	% of total	Length of rivers (10^3 km)	% of total	Population exposed (millions)
Afghanistan	68.4	85.8	55.0	69.1	Amoxicillin	5.6	7.0	2.2	2.7	3.3
Albania	3.3	40.6	2.0	24.5	Amoxicillin	0.1	1.1	< 0.1	0.2	0.1
Algeria	42.7	45.3	38.8	41.2	Amoxicillin	9.6	10.1	9.5	10.0	7.2
Andorra	0.1	89.0	0.1	75.9	Amoxicillin	< 0.1	16.8	0.0	0.0	0.0
Angola	45.3	20.0	11.8	5.2	Amoxicillin	1.0	0.4	0.5	0.2	1.1
Anguilla	< 0.1	100.0	< 0.1	100.0	Penicillin V	0.0	0.0	0.0	0.0	0.0
Antigua and Barbuda	< 0.1	67.1	< 0.1	67.1	Amoxicillin	< 0.1	11.6	< 0.1	2.7	< 0.1
Argentina	44.4	13.5	28.9	8.8	Amoxicillin	4.4	1.3	3.1	0.9	2.8
Armenia	2.4	59.0	1.5	38.3	Amoxicillin	0.3	7.0	0.2	5.3	0.6
Aruba	0.0	0.0	0.0	0.0	-	0.0	0.0	0.0	0.0	0.0
Australia	48.3	7.1	30.9	4.5	Cefalexin	5.4	0.8	2.6	0.4	2.8
Austria	9.4	41.1	3.1	13.6	Penicillin V	0.0	0.0	0.0	0.0	0.0
Azerbaijan	12.9	96.3	8.9	66.4	Amoxicillin	1.2	9.2	0.3	2.4	0.3
Bahamas	0.1	4.0	< 0.1	2.8	Amoxicillin	< 0.1	0.3	0.0	0.0	0.0
Bahrain	0.0	0.0	0.0	0.0	-	0.0	0.0	0.0	0.0	0.0
Bangladesh	54.7	96.8	50.4	89.1	Ciprofloxacin	2.8	5.0	1.8	3.1	18.3
Barbados	0.1	100.0	0.1	100.0	Amoxicillin	< 0.1	13.4	< 0.1	2.0	< 0.1
Belarus	21.4	49.7	7.3	17.0	Ceftriaxone	0.2	0.4	< 0.1	0.1	0.6
Belgium	7.1	90.0	5.9	75.4	Amoxicillin	0.1	0.8	< 0.1	0.2	< 0.1
Belize	0.2	3.6	0.0	0.4	Amoxicillin	0.0	0.0	0.0	0.0	0.0
Benin	11.1	51.2	3.8	17.6	Amoxicillin	0.1	0.3	< 0.1	< 0.1	0.2
Bhutan	1.8	17.7	0.4	4.2	Amoxicillin	0.0	0.0	0.0	0.0	0.0
Bolivia	26.6	12.6	9.5	4.5	Amoxicillin	0.8	0.4	0.4	0.2	0.4
Bonaire, Saint Eustatius and Saba	< 0.1	31.3	< 0.1	31.3	Amoxicillin	0.0	0.0	0.0	0.0	0.0
Bosnia and Herzegovina	6.4	48.4	2.6	19.4	Ciprofloxacin	< 0.1	0.1	0.0	0.0	0.0
Botswana	10.8	40.0	8.3	30.6	Ceftriaxone	2.9	10.9	1.3	4.9	0.1
Brazil	211.0	8.8	109.0	4.5	Ciprofloxacin	7.2	0.3	3.7	0.2	33.9
British Virgin Islands	0.0	0.0	0.0	0.0	-	0.0	0.0	0.0	0.0	0.0
Brunei	0.2	9.9	0.1	5.3	Amoxicillin	0.0	0.0	0.0	0.0	0.0
Bulgaria	17.0	74.1	7.6	33.3	Ceftriaxone	0.2	0.8	0.1	0.2	0.1
Burkina Faso	23.9	91.1	16.6	63.5	Amoxicillin	0.8	3.0	0.4	1.6	0.4
Burundi	5.6	99.1	5.5	96.9	Amoxicillin	0.1	2.6	< 0.1	0.8	0.6
Cote d'Ivoire	17.8	25.4	5.9	8.3	Amoxicillin	0.3	0.4	0.1	0.1	1.1
Cambodia	20.1	34.1	8.0	13.5	Amoxicillin	0.1	0.1	0.0	0.0	0.0
Cameroon	14.2	11.3	8.6	6.9	Amoxicillin	0.3	0.2	0.1	0.0	1.4
Canada	16.9	0.8	5.7	0.3	Amoxicillin	0.4	0.0	0.1	0.0	0.7
Cayman Islands	< 0.1	12.0	< 0.1	12.0	Amoxicillin	0.0	0.0	0.0	0.0	0.0

Central African Republic	5.7	4.5	1.0	0.8	Amoxicillin	0.0	0.0	< 0.1	< 0.1	0.6
Chad	35.5	41.7	17.3	20.4	Amoxicillin	3.1	3.6	1.8	2.2	0.1
Chile	16.8	12.0	11.2	8.0	Amoxicillin	1.3	0.9	1.6	1.1	5.9
China	669.0	46.4	353.0	24.5	Amoxicillin	74.4	5.2	24.6	1.7	89.8
Clipperton Island	0.0	0.0	0.0	0.0	-	0.0	0.0	0.0	0.0	0.0
Colombia	16.0	3.4	5.9	1.3	Amoxicillin	0.2	< 0.1	0.2	< 0.1	0.3
Comoros	0.5	99.7	0.4	79.5	Amoxicillin	0.0	0.0	0.0	0.0	0.0
Costa Rica	1.4	6.4	0.4	1.9	Ciprofloxacin	0.0	0.0	0.0	0.0	0.0
Croatia	7.4	55.2	4.5	33.4	Amoxicillin	0.2	1.2	0.1	0.7	0.4
Cuba	17.3	68.8	7.3	29.0	Amoxicillin	0.3	1.2	0.1	0.3	0.1
Curaçao	0.0	0.0	0.0	0.0	-	0.0	0.0	0.0	0.0	0.0
Cyprus	1.4	88.7	1.1	69.2	Amoxicillin	0.3	21.3	0.2	11.6	0.2
Czech Republic	13.2	77.6	10.7	62.5	Penicillin V	0.0	0.0	0.0	0.0	0.0
Denmark	6.4	65.9	5.7	58.4	Amoxicillin	0.0	0.0	0.0	0.0	0.0
Djibouti	1.3	99.4	0.8	64.1	Penicillin V	0.3	24.9	0.3	19.8	0.1
Dominica	0.1	37.3	< 0.1	10.0	Amoxicillin	0.0	0.0	0.0	0.0	0.0
Dominican Republic	4.4	38.4	1.9	16.5	Amoxicillin	0.1	0.7	< 0.1	0.1	0.3
DR Congo	102.0	18.4	33.2	6.0	Ciprofloxacin	1.2	0.2	0.4	0.1	7.6
East Timor	2.2	74.1	1.1	37.9	Amoxicillin	< 0.1	0.8	< 0.1	0.5	0.1
Ecuador	12.8	13.5	4.3	4.6	Ciprofloxacin	0.6	0.6	0.3	0.3	0.7
Egypt	3.5	58.7	3.2	52.5	Cefadroxil	0.9	15.4	0.7	11.3	2.7
El Salvador	5.1	75.4	2.1	30.9	Ciprofloxacin	0.1	1.1	0.0	0.0	0.0
Equatorial Guinea	0.5	5.0	0.2	2.3	Amoxicillin	0.0	0.0	0.0	0.0	0.0
Eritrea	12.5	85.7	9.1	62.1	Amoxicillin	2.7	18.3	0.9	6.0	0.3
Estonia	0.4	3.6	0.1	1.2	Amoxicillin	< 0.1	0.1	< 0.1	0.1	< 0.1
Ethiopia	162.0	87.4	125.0	67.3	Amoxicillin	18.7	10.1	5.4	2.9	7.2
Falkland Islands	0.0	0.0	0.0	0.0	-	0.0	0.0	0.0	0.0	0.0
Faroe Islands	< 0.1	6.7	< 0.1	3.1	Amoxicillin	0.0	0.0	0.0	0.0	0.0
Fiji	0.2	4.2	0.1	1.4	Amoxicillin	0.0	0.0	0.0	0.0	0.0
Finland	6.2	8.1	2.0	2.6	Penicillin V	0.0	0.0	0.0	0.0	0.0
France	55.3	39.6	16.0	11.5	Amoxicillin	0.0	0.0	0.0	0.0	0.0
French Guiana	0.2	0.6	0.1	0.4	Amoxicillin	0.0	0.0	0.0	0.0	0.0
French Southern Territories	0.0	0.0	0.0	0.0		0.0	0.0	0.0	0.0	0.0
Gabon	1.8	2.1	0.2	0.3	Amoxicillin	0.1	0.1	0.0	0.0	0.0
Gambia	1.2	87.2	0.9	62.9	Amoxicillin	0.1	9.0	0.1	6.6	0.5
Georgia	3.6	18.7	1.1	5.8	Amoxicillin	0.1	0.3	0.0	0.1	0.1
Germany	70.1	83.2	31.8	37.7	Penicillin V	0.2	0.2	0.1	0.1	0.4
Ghana	37.8	78.4	17.5	36.3	Amoxicillin	0.9	2.0	0.4	0.9	3.6
Gibraltar	0.0	0.0	0.0	0.0		0.0	0.0	0.0	0.0	0.0
Greece	14.5	61.6	8.8	37.5	Amoxicillin	0.1	0.4	0.2	0.6	0.6
Greenland	0.0	0.0	0.0	0.0	Amoxicillin	0.0	0.0	0.0	0.0	0.0
Grenada	< 0.1	100.0	< 0.1	100.0	Amoxicillin	< 0.1	39.7	0.0	0.0	0.0
Guadeloupe	0.3	96.5	0.3	85.3	Amoxicillin	0.0	0.0	0.0	0.0	0.0
Guam	0.0	0.0	0.0	0.0	Amoxicillin	0.0	0.0	0.0	0.0	0.0
Guatemala	13.1	34.9	4.1	10.8	Ciprofloxacin	0.2	0.5	< 0.1	< 0.1	0.3
Guernsey	< 0.1	100.0	< 0.1	100.0	Amoxicillin	0.0	0.0	0.0	0.0	0.0
Guinea	6.5	9.5	2.4	3.4	Amoxicillin	0.2	0.3	< 0.1	< 0.1	0.3
Guinea-Bissau	4.8	56.4	0.9	10.3	Amoxicillin	0.1	0.9	< 0.1	0.5	0.3
Guyana	0.3	0.4	0.1	0.2	Amoxicillin	0.0	0.0	0.0	0.0	0.0
Haiti	5.6	93.8	3.6	59.4	Amoxicillin	0.1	1.9	0.1	0.9	1.4
Honduras	6.6	19.8	1.5	4.5	Ciprofloxacin	0.1	0.3	0.0	0.0	0.0
Hong Kong	0.1	71.9	0.1	52.2	Amoxicillin	< 0.1	19.3	< 0.1	9.4	1.1
Hungary	11.7	70.2	4.6	27.4	Ceftriaxone	< 0.1	0.2	< 0.1	0.1	< 0.1

Iceland	0.1	0.2	< 0.1	0.1	Amoxicillin	0.0	0.0	0.0	0.0	0.0
India	677.0	87.2	623.0	80.3	Cefixime	274.0	35.3	111.0	14.3	315.0
Indonesia	101.0	14.4	62.7	8.9	Amoxicillin	1.6	0.2	0.4	0.1	5.0
Iran	183.0	90.7	147.0	72.7	Amoxicillin	29.4	14.5	10.8	5.3	9.3
Iraq	31.8	87.3	29.4	80.9	Amoxicillin	9.7	26.6	5.0	13.8	3.7
Ireland	1.2	5.7	0.4	1.8	Penicillin V	0.0	0.0	0.0	0.0	0.0
Isle of Man	0.1	48.2	< 0.1	22.4	Amoxicillin	0.0	0.0	0.0	0.0	0.0
Israel	2.9	93.3	2.8	90.5	Amoxicillin	1.9	62.0	1.2	36.9	3.3
Italy	31.9	42.3	19.7	26.2	Ceftriaxone	1.3	1.8	0.4	0.6	2.0
Jamaica	2.3	87.7	1.3	49.9	Amoxicillin	0.1	4.0	< 0.1	1.5	< 0.1
Japan	19.8	15.8	7.0	5.6	Ceftriaxone	0.1	0.1	0.0	0.0	0.4
Jersey	< 0.1	100.0	< 0.1	100.0	Amoxicillin	< 0.1	100.0	< 0.1	52.6	< 0.1
Jordan	2.3	87.0	2.3	85.6	Amoxicillin	1.7	64.0	1.0	39.0	2.3
Kazakhstan	73.2	48.0	57.6	37.8	Ceftriaxone	3.7	2.5	2.3	1.5	1.6
Kenya	58.3	66.0	36.2	41.0	Amoxicillin	3.3	3.7	1.1	1.3	4.1
Kiribati	0.0	0.0	0.0	0.0	-	0.0	0.0	0.0	0.0	0.0
Kosovo	2.5	86.2	1.3	45.3	Amoxicillin	0.0	0.0	0.0	0.0	0.0
Kuwait	0.2	100.0	0.2	100.0	Amoxicillin	0.1	29.5	0.1	29.5	0.0
Kyrgyzstan	15.4	44.2	7.4	21.4	Amoxicillin	1.1	3.3	0.4	1.2	0.3
Laos	12.9	17.9	2.7	3.8	Amoxicillin	0.1	0.1	< 0.1	0.1	0.2
Latvia	1.2	7.5	0.3	2.0	Amoxicillin	0.0	0.0	0.0	0.0	0.0
Lebanon	2.6	96.2	2.3	87.7	Amoxicillin	1.0	38.2	0.7	25.2	1.9
Lesotho	3.3	65.8	1.3	26.4	Amoxicillin	0.1	1.5	0.0	0.0	0.0
Liberia	2.8	7.2	0.4	1.0	Amoxicillin	0.1	0.2	< 0.1	0.1	0.3
Libya	17.9	41.9	14.0	32.9	Ceftriaxone	5.7	13.3	3.3	7.7	0.7
Liechtenstein	< 0.1	75.1	< 0.1	75.1	Amoxicillin	0.0	0.0	0.0	0.0	0.0
Lithuania	10.1	65.2	3.4	21.6	Amoxicillin	0.0	0.0	0.0	0.0	0.0
Luxembourg	0.6	94.2	0.6	84.6	Amoxicillin	0.0	0.0	0.0	0.0	0.0
Macao	0.0	0.0	0.0	0.0	-	0.0	0.0	0.0	0.0	0.0
Macedonia	4.0	75.1	2.5	46.4	Amoxicillin	0.2	3.4	< 0.1	0.6	0.1
Madagascar	27.0	15.7	6.8	4.0	Amoxicillin	0.3	0.2	0.1	0.1	1.2
Malawi	17.8	87.1	15.8	77.1	Amoxicillin	2.6	12.5	0.5	2.6	1.8
Malaysia	4.7	3.7	2.0	1.6	Amoxicillin	0.0	0.0	0.0	0.0	0.0
Mali	37.7	44.6	17.3	20.4	Amoxicillin	1.7	2.0	1.1	1.3	1.2
Malta	0.0	0.0	0.0	0.0	-	0.0	0.0	0.0	0.0	0.0
Marshall Islands	0.0	0.0	0.0	0.0	-	0.0	0.0	0.0	0.0	0.0
Martinique	0.2	64.7	0.2	57.5	Amoxicillin	0.0	0.0	0.0	0.0	0.0
Mauritania	15.8	47.7	11.2	33.7	Amoxicillin	3.0	8.9	1.4	4.4	0.1
Mayotte	< 0.1	34.8	< 0.1	34.8	Amoxicillin	0.0	0.0	0.0	0.0	0.0
Mexico	95.2	35.2	50.5	18.7	Ciprofloxacin	9.2	3.4	4.5	1.6	12.3
Micronesia	0.0	0.0	0.0	0.0	-	0.0	0.0	0.0	0.0	0.0
Moldova	3.6	58.9	1.9	30.7	Ceftriaxone	0.1	0.9	< 0.1	0.2	0.1
Monaco	0.0	0.0	0.0	0.0	-	0.0	0.0	0.0	0.0	0.0
Mongolia	7.6	10.8	4.1	5.8	Ceftriaxone	1.7	2.3	0.7	1.0	0.2
Montenegro	0.8	17.7	0.2	5.2	Amoxicillin	0.0	0.0	0.0	0.0	0.0
Montserrat	0.0	0.0	0.0	0.0	-	0.0	0.0	0.0	0.0	0.0
Morocco	40.0	75.8	35.6	67.5	Amoxicillin	2.3	4.4	3.0	5.7	6.2
Mozambique	72.9	44.8	24.2	14.9	Amoxicillin	1.3	0.8	0.7	0.4	1.7
Myanmar	80.4	32.3	38.6	15.5	Amoxicillin	0.9	0.3	0.3	0.1	2.9
Namibia	14.5	40.6	8.2	22.9	Ceftriaxone	2.5	6.9	1.5	4.1	0.1
Nauru	< 0.1	100.0	< 0.1	61.3	Amoxicillin	0.0	0.0	0.0	0.0	0.0
Nepal	29.0	58.9	17.5	35.6	Amoxicillin	1.7	3.4	0.2	0.5	1.9
Netherlands	2.6	30.7	1.2	14.3	Amoxicillin	< 0.1	0.3	< 0.1	0.3	0.2
New Caledonia	0.1	2.4	< 0.1	0.8	Amoxicillin	< 0.1	0.1	0.0	0.0	0.0
New Zealand	0.5	0.6	0.3	0.4	Amoxicillin	0.0	0.0	0.0	0.0	0.0
Nicaragua	5.4	12.8	1.9	4.5	Ciprofloxacin	0.1	0.3	0.0	0.0	0.0
Niger	21.9	44.3	16.7	33.8	Amoxicillin	5.4	10.8	2.7	5.5	0.7
Nigeria	174.0	86.2	120.0	59.5	Amoxicillin	15.7	7.8	4.7	2.3	18.2

Norfolk Island	0.0	0.0	0.0	0.0	-	0.0	0.0	0.0	0.0	0.0
North Korea	23.9	80.7	12.9	43.4	Amoxicillin	1.1	3.5	0.3	1.2	1.7
Northern Mariana Islands	< 0.1	3.6	< 0.1	3.6	Amoxicillin	0.0	0.0	0.0	0.0	0.0
Norway	3.7	4.0	3.1	3.4	Penicillin V	0.1	0.1	0.0	0.0	0.0
Oman	10.9	78.0	10.7	76.1	Penicillin V	8.4	59.6	5.8	41.2	0.6
Pakistan	91.2	89.8	77.6	76.4	Ciprofloxacin	31.4	31.0	17.2	17.0	23.0
Palau	0.0	0.0	0.0	0.0	-	0.0	0.0	0.0	0.0	0.0
Palestine	1.2	100.0	1.2	96.7	Amoxicillin	0.9	71.9	0.5	43.9	1.6
Panama	0.7	2.7	0.3	1.0	Ciprofloxacin	0.0	0.0	0.0	0.0	0.0
Papua New Guinea	0.3	0.2	0.1	< 0.1	Amoxicillin	0.0	0.0	0.0	0.0	0.0
Paraguay	5.1	8.1	2.0	3.1	Amoxicillin	0.3	0.4	0.1	0.1	1.1
Peru	17.4	4.1	5.0	1.2	Ceftriaxone	0.7	0.2	0.4	0.1	0.2
Philippines	35.2	32.9	18.5	17.2	Amoxicillin	1.7	1.6	0.3	0.2	8.3
Poland	35.7	55.1	12.0	18.5	Amoxicillin	0.0	0.0	< 0.1	0.1	0.2
Portugal	7.4	34.7	3.4	15.7	Amoxicillin	0.1	0.5	0.1	0.3	0.1
Puerto Rico	2.1	90.8	1.8	77.4	Ciprofloxacin	0.1	2.6	0.0	0.0	0.0
Qatar	0.0	0.0	0.0	0.0	-	0.0	0.0	0.0	0.0	0.0
Republic of Congo	0.2	0.2	0.1	0.2	Amoxicillin	0.0	0.0	< 0.1	< 0.1	0.5
Romania	44.2	85.0	25.4	48.8	Amoxicillin	0.4	0.7	0.1	0.3	0.6
Russia	285.0	8.3	217.0	6.3	Ceftriaxone	7.2	0.2	3.2	0.1	11.2
Rwanda	4.7	94.0	4.6	91.8	Amoxicillin	0.2	3.3	< 0.1	0.5	0.3
Saint Kitts and Nevis	0.0	0.0	0.0	0.0	-	0.0	0.0	0.0	0.0	0.0
Saint Lucia	0.1	94.3	0.1	60.6	Amoxicillin	< 0.1	1.0	0.0	0.0	0.0
Saint Pierre and Miquelon	0.0	0.0	0.0	0.0	-	0.0	0.0	0.0	0.0	0.0
Saint Vincent and the Grenadines	0.1	95.3	< 0.1	55.1	Amoxicillin	< 0.1	1.6	0.0	0.0	0.0
Saint-Barthelemy	0.0	0.0	0.0	0.0	-	0.0	0.0	0.0	0.0	0.0
Saint-Martin	0.0	0.0	0.0	0.0	-	0.0	0.0	0.0	0.0	0.0
San Marino	< 0.1	100.0	< 0.1	100.0	Amoxicillin	0.0	0.0	0.0	0.0	0.0
Sao Tome and Principe	0.1	53.7	0.1	35.4	Amoxicillin	0.0	0.0	0.0	0.0	0.0
Saudi Arabia	53.7	75.2	49.5	69.3	Amoxicillin	22.6	31.7	16.0	22.4	2.2
Senegal	11.5	62.2	6.2	33.3	Amoxicillin	0.4	2.2	0.2	1.0	0.1
Serbia	12.6	81.5	9.6	61.8	Cefalexin	0.3	2.2	0.1	0.8	0.5
Seychelles	0.0	0.0	0.0	0.0	-	0.0	0.0	0.0	0.0	0.0
Sierra Leone	9.0	28.8	1.9	6.1	Amoxicillin	0.1	0.5	0.1	0.2	0.6
Singapore	0.1	32.8	0.0	29.1	Amoxicillin	0.0	9.4	0.0	5.6	0.4
Sint Maarten	0.0	0.0	0.0	0.0	-	0.0	0.0	0.0	0.0	0.0
Slovakia	8.6	78.1	6.0	54.4	Penicillin V	0.1	0.6	< 0.1	0.4	< 0.1
Slovenia	1.7	29.1	0.8	14.3	Penicillin V	0.0	0.0	0.0	0.0	0.0
Solomon Islands	0.0	0.2	0.0	0.1	Amoxicillin	0.0	0.0	0.0	0.0	0.0
Somalia	41.4	98.1	33.1	78.3	Ceftriaxone	9.6	22.7	5.4	12.7	0.6
South Africa	60.3	56.1	41.5	38.7	Amoxicillin	5.6	5.2	4.5	4.2	6.2
South Korea	19.1	74.5	14.5	56.4	Amoxicillin	1.2	4.8	0.4	1.5	3.3
South Sudan	42.6	57.4	18.2	24.6	Amoxicillin	0.9	1.2	0.2	0.3	< 0.1
Spain	45.1	49.8	30.4	33.5	Amoxicillin	2.2	2.5	1.2	1.3	3.1
Spratly islands	0.0	0.0	0.0	0.0	-	0.0	0.0	0.0	0.0	0.0
Sri Lanka	12.0	63.8	8.0	42.7	Ciprofloxacin	0.1	0.3	< 0.1	0.2	0.5
Sudan	80.2	80.0	59.2	59.1	Ceftriaxone	16.5	16.5	5.3	5.3	1.1
Suriname	0.3	0.5	0.2	0.4	Amoxicillin	< 0.1	0.1	0.0	0.0	0.0
Svalbard and Jan Mayen	0.0	0.0	0.0	0.0	-	0.0	0.0	0.0	0.0	0.0

Swaziland	3.2	93.0	2.0	56.7	Amoxicillin	< 0.1	1.0	< 0.1	0.3	< 0.1
Sweden	4.3	4.2	3.6	3.5	Penicillin V	0.0	0.0	0.0	0.0	0.0
Switzerland	0.5	4.0	0.1	0.9	Amoxicillin	0.0	0.0	0.0	0.0	0.0
Syria	11.5	96.8	10.9	91.1	Amoxicillin	3.4	28.3	1.4	11.5	4.5
Taiwan	5.2	39.3	3.8	29.1	Amoxicillin	0.3	2.7	0.1	1.1	1.4
Tajikistan	10.4	36.8	6.2	22.1	Amoxicillin	0.7	2.6	0.2	0.7	0.3
Tanzania	84.9	52.6	47.3	29.3	Amoxicillin	3.9	2.4	1.4	0.8	5.1
Thailand	97.0	71.6	64.7	47.8	Amoxicillin	2.0	1.4	0.9	0.7	8.2
Togo	4.9	38.8	2.0	15.8	Amoxicillin	0.1	0.6	< 0.1	0.3	0.6
Trinidad and Tobago	0.9	69.0	0.6	47.1	Amoxicillin	< 0.1	2.7	< 0.1	0.1	< 0.1
Tunisia	11.7	83.0	11.3	80.2	Amoxicillin	3.2	22.7	3.0	21.5	1.5
Turkey	127.0	84.0	93.7	61.9	Amoxicillin	12.9	8.5	8.9	5.9	16.2
Turkmenistan	13.9	92.9	11.6	77.7	Ceftriaxone	4.8	31.9	3.2	21.7	0.4
Turks and Caicos Islands	0.0	0.0	0.0	0.0	-	0.0	0.0	0.0	0.0	0.0
Tuvalu	0.0	0.0	0.0	0.0	-	0.0	0.0	0.0	0.0	0.0
Uganda	30.2	88.8	22.4	65.9	Amoxicillin	1.0	2.8	0.2	0.5	2.5
Ukraine	72.9	74.2	65.1	66.3	Ceftriaxone	1.2	1.2	0.6	0.6	1.3
United Arab Emirates	0.9	97.4	0.9	97.4	Amoxicillin	0.9	89.3	0.8	81.8	0.8
United Kingdom	14.2	20.4	7.8	11.3	Penicillin V	0.1	0.1	0.0	0.0	0.0
United States	260.0	14.6	97.6	5.5	Amoxicillin	13.2	0.7	7.2	0.4	11.8
United States Minor Outlying Islands	0.0	0.0	0.0	0.0	-	0.0	0.0	0.0	0.0	0.0
Uruguay	1.1	2.2	0.7	1.4	Amoxicillin	0.1	0.1	< 0.1	0.1	< 0.1
Uzbekistan	14.7	79.6	11.2	60.7	Amoxicillin	4.0	21.8	1.4	7.3	0.8
Vanuatu	0.0	0.4	0.0	0.3	Amoxicillin	0.0	0.0	0.0	0.0	0.0
Vatican City	0.0	0.0	0.0	0.0	-	0.0	0.0	0.0	0.0	0.0
Venezuela	52.0	18.2	34.5	12.1	Ampicillin	1.7	0.6	1.0	0.4	3.3
Vietnam	96.5	88.0	52.1	47.5	Cefalexin	12.8	11.7	2.1	2.0	16.9
Virgin Islands	< 0.1	100.0	< 0.1	72.3	Amoxicillin	0.0	0.0	0.0	0.0	0.0
Western Sahara	0.2	1.8	0.1	1.2	Ceftriaxone	0.1	0.6	0.1	0.6	< 0.1
Yemen	16.3	70.9	14.3	61.9	Amoxicillin	9.1	39.5	5.2	22.7	5.3
Zambia	41.5	31.0	15.3	11.4	Amoxicillin	0.9	0.7	0.4	0.3	1.4
Zimbabwe	47.1	85.7	26.1	47.4	Amoxicillin	0.7	1.3	0.4	0.6	1.5
**Global**	**5,980.0**	**25.1**	**3,810.0**	**16.0**	**Amoxicillin**	**723.0**	**3.0**	**324.0**	**1.4**	**751.0**

SI References

Sample References:

1. Wilkinson, J. L. et al. Pharmaceutical pollution of the world's rivers. Proceedings of the National Academy of Sciences 119, e2113947119, doi:10.1073/pnas.2113947119 (2022).
2. NORMAN Network. NORMAN Database System, <https://www.norman-network.com/nds/> (2024).
3. Umweltbundesamt. PHARMS-UBA - Pharmaceuticals in the environment, <https://www.umweltbundesamt.de/en/database-pharmaceuticals-in-the-environment-0 > (2024).
4. Adachi, F., Yamamoto, A., Takakura, K. & Kawahara, R. Occurrence of fluoroquinolones and fluoroquinolone-resistance genes in the aquatic environment. Sci Total Environ 444, 508-514, doi:10.1016/j.scitotenv.2012.11.077 (2013).
5. Agunbiade, F. O. & Moodley, B. Occurrence and distribution pattern of acidic pharmaceuticals in surface water, wastewater, and sediment of the Msunduzi River, Kwazulu-Natal, South Africa. Environ Toxicol Chem 35, 36-46, doi:10.1002/etc.3144 (2016).
6. Arikan, O. A., Rice, C. & Codling, E. Occurrence of antibiotics and hormones in a major agricultural watershed. Desalination 226, 121-133, doi:10.1016/j.desal.2007.01.238 (2008).
7. Arsand, J. B. et al. Presence of antibiotic resistance genes and its association with antibiotic occurrence in Dilúvio River in southern Brazil. Sci Total Environ 738, 139781, doi:10.1016/j.scitotenv.2020.139781 (2020).
8. Aydin, E. & Talinli, I. Analysis, occurrence and fate of commonly used pharmaceuticals and hormones in the Buyukcekmece Watershed, Turkey. Chemosphere 90, 2004-2012, doi:10.1016/j.chemosphere.2012.10.074 (2013).
9. Bagnis, S. et al. Characterization of the Nairobi River catchment impact zone and occurrence of pharmaceuticals: Implications for an impact zone inclusive environmental risk assessment. Science of The Total Environment 703, 134925, doi:10.1016/j.scitotenv.2019.134925 (2020).
10. Barber, L. B. et al. Lagrangian sampling of wastewater treatment plant effluent in Boulder Creek, Colorado, and Fourmile Creek, Iowa, during the summer of 2003 and spring of 2005— Hydrological and water-quality data. Report No. 2011-1054, 84 (Reston, VA, 2011).
11. Batt, A. L., Bruce, I. B. & Aga, D. S. Evaluating the vulnerability of surface waters to antibiotic contamination from varying wastewater treatment plant discharges. Environ Pollut 142, 295-302, doi:10.1016/j.envpol.2005.10.010 (2006).
12. Bendz, D., Paxéus, N. A., Ginn, T. R. & Loge, F. J. Occurrence and fate of pharmaceutically active compounds in the environment, a case study: Höje River in Sweden. J Hazard Mater 122, 195-204, doi:10.1016/j.jhazmat.2005.03.012 (2005).
13. Böger, B. et al. Occurrence of antibiotics and antibiotic resistant bacteria in subtropical urban rivers in Brazil. Journal of Hazardous Materials 402, 123448, doi:10.1016/j.jhazmat.2020.123448 (2021).
14. Calamari, D., Zuccato, E., Castiglioni, S., Bagnati, R. & Fanelli, R. Strategic Survey of Therapeutic Drugs in the Rivers Po and Lambro in Northern Italy. Environmental Science & Technology 37, 1241-1248, doi:10.1021/es020158e (2003).
15. Camacho-Muñoz, D., Martín, J., Santos, J. L., Aparicio, I. & Alonso, E. Occurrence, temporal evolution and risk assessment of pharmaceutically active compounds in Doñana Park (Spain). J Hazard Mater 183, 602-608, doi:10.1016/j.jhazmat.2010.07.067 (2010).
16. Carlson, J. C. et al. Presence and hazards of nutrients and emerging organic micropollutants from sewage lagoon discharges into Dead Horse Creek, Manitoba, Canada. Science of The Total Environment 445-446, 64-78, doi:10.1016/j.scitotenv.2012.11.100 (2013).

17. Chang, X. et al. Determination of antibiotics in sewage from hospitals, nursery and slaughter house, wastewater treatment plant and source water in Chongqing region of Three Gorge Reservoir in China. Environmental Pollution 158, 1444-1450, doi:10.1016/j.envpol.2009.12.034 (2010).
18. Chau, H. T. C. et al. Occurrence of 1153 organic micropollutants in the aquatic environment of Vietnam. Environ Sci Pollut Res Int 25, 7147-7156, doi:10.1007/s11356-015-5060-z (2018).
19. Chaves, M. d. J. S. et al. Pharmaceuticals and personal care products in a Brazilian wetland of international importance: Occurrence and environmental risk assessment. Science of The Total Environment 734, 139374, doi:10.1016/j.scitotenv.2020.139374 (2020).
20. Chitescu, C. L., Kaklamanos, G., Nicolau, A. I. & Stolker, A. A. M. High sensitive multiresidue analysis of pharmaceuticals and antifungals in surface water using U-HPLC-Q-Exactive Orbitrap HRMS. Application to the Danube river basin on the Romanian territory. Science of The Total Environment 532, 501-511, doi:10.1016/j.scitotenv.2015.06.010 (2015).
21. Choi, K. et al. Seasonal variations of several pharmaceutical residues in surface water and sewage treatment plants of Han River, Korea. Science of The Total Environment 405, 120-128, doi:10.1016/j.scitotenv.2008.06.038 (2008).
22. Deng, W. J., Li, N. & Ying, G. G. Antibiotic distribution, risk assessment, and microbial diversity in river water and sediment in Hong Kong. Environ Geochem Health 40, 2191-2203, doi:10.1007/s10653-018-0092-1 (2018).
23. Dinh, Q. T. et al. Measurement of trace levels of antibiotics in river water using on-line enrichment and triple-quadrupole LC-MS/MS. Talanta 85, 1238-1245, doi:10.1016/j.talanta.2011.05.013 (2011).
24. Ekberg, M. P. & Pletsch, B. A. Pharmaceuticals and Personal Care Products (PPCPs) in the Streams and Aquifers of the Great Miami River Basin. 38 (2011).
25. Feitosa-Felizzola, J. & Chiron, S. Occurrence and distribution of selected antibiotics in a small Mediterranean stream (Arc River, Southern France). Journal of Hydrology 364, 50-57, doi:10.1016/j.jhydrol.2008.10.006 (2009).
26. Fernandes, M. J. et al. Antibiotics and antidepressants occurrence in surface waters and sediments collected in the north of Portugal. Chemosphere 239, 124729, doi:10.1016/j.chemosphere.2019.124729 (2020).
27. Fick, J., Lindberg, R., Kaj, L. & Brorström-Lundén, E. Results from the Swedish National Screening Programme 2010. (2011).
28. Fick, J. et al. Contamination of surface, ground, and drinking water from pharmaceutical production. Environ Toxicol Chem 28, 2522-2527, doi:10.1897/09-073.1 (2009).
29. Finnegan, D. P., Simonson, L. A. & Meyer, M. T. Occurrence of antibiotic compounds in source water and finished drinking water from the upper Scioto River Basin, Ohio, 2005-6. 16 (U. S. Geological Survey,, 2010).
30. Fonseca, E. et al. Occurrence and ecological risks of pharmaceuticals in a Mediterranean river in Eastern Spain. Environment International 144, 106004, doi:10.1016/j.envint.2020.106004 (2020).
31. Hanna, N. et al. Monitoring of Water Quality, Antibiotic Residues, and Antibiotic-Resistant Escherichia coli in the Kshipra River in India over a 3-Year Period. Int J Environ Res Public Health 17, doi:10.3390/ijerph17217706 (2020).
32. Joshua, D. I. et al. First report of pharmaceuticals and personal care products in two tropical rivers of southwestern India. Environmental Monitoring and Assessment 192, 529, doi:10.1007/s10661-020-08480-2 (2020).
33. K'Oreje, K. O. et al. From multi-residue screening to target analysis of pharmaceuticals in water: Development of a new approach based on magnetic sector mass spectrometry and application in the Nairobi River basin, Kenya. Science of The Total Environment 437, 153-164, doi:10.1016/j.scitotenv.2012.07.052 (2012).
34. Kairigo, P., Ngumba, E., Sundberg, L.-R., Gachanja, A. & Tuhkanen, T. Occurrence of antibiotics and risk of antibiotic resistance evolution in selected Kenyan wastewaters,

surface waters and sediments. Science of The Total Environment 720, 137580, doi:10.1016/j.scitotenv.2020.137580 (2020).
35. Kandie, F. J. et al. Occurrence and risk assessment of organic micropollutants in freshwater systems within the Lake Victoria South Basin, Kenya. Science of The Total Environment 714, 136748, doi:10.1016/j.scitotenv.2020.136748 (2020).
36. Kasprzyk-Hordern, B., Dinsdale, R. M. & Guwy, A. J. Multi-residue method for the determination of basic/neutral pharmaceuticals and illicit drugs in surface water by solid-phase extraction and ultra performance liquid chromatography-positive electrospray ionisation tandem mass spectrometry. J Chromatogr A 1161, 132-145, doi:10.1016/j.chroma.2007.05.074 (2007).
37. Kasprzyk-Hordern, B., Dinsdale, R. M. & Guwy, A. J. The occurrence of pharmaceuticals, personal care products, endocrine disruptors and illicit drugs in surface water in South Wales, UK. Water Research 42, 3498-3518, doi:10.1016/j.watres.2008.04.026 (2008).
38. Khan, G. A., Berglund, B., Khan, K. M., Lindgren, P.-E. & Fick, J. Occurrence and abundance of antibiotics and resistance genes in rivers, canal and near drug formulation facilities–a study in Pakistan. PloS one 8, e62712 (2013).
39. Khan, G. A., Lindberg, R., Grabic, R. & Fick, J. The development and application of a system for simultaneously determining anti-infectives and nasal decongestants using on-line solid-phase extraction and liquid chromatography–tandem mass spectrometry. Journal of Pharmaceutical and Biomedical Analysis 66, 24-32, doi:10.1016/j.jpba.2012.02.011 (2012).
40. Kim, S.-C. & Carlson, K. Temporal and Spatial Trends in the Occurrence of Human and Veterinary Antibiotics in Aqueous and River Sediment Matrices. Environmental Science & Technology 41, 50-57, doi:10.1021/es060737+ (2007).
41. Kunkel, U. & Radke, M. Fate of pharmaceuticals in rivers: Deriving a benchmark dataset at favorable attenuation conditions. Water Research 46, 5551-5565, doi:10.1016/j.watres.2012.07.033 (2012).
42. Li, Z., Sobek, A. & Radke, M. Fate of Pharmaceuticals and Their Transformation Products in Four Small European Rivers Receiving Treated Wastewater. Environmental Science & Technology 50, 5614-5621, doi:10.1021/acs.est.5b06327 (2016).
43. Locatelli, M. A. F., Sodré, F. F. & Jardim, W. F. Determination of Antibiotics in Brazilian Surface Waters Using Liquid Chromatography–Electrospray Tandem Mass Spectrometry. Archives of Environmental Contamination and Toxicology 60, 385-393, doi:10.1007/s00244-010-9550-1 (2011).
44. Loper, C. A. et al. Concentrations of selected pharmaceuticals and antibiotics in south-central Pennsylvania waters, March through September 2006. Report No. 300, (Reston, VA, 2007).
45. López-Serna, R., Petrović, M. & Barceló, D. Development of a fast instrumental method for the analysis of pharmaceuticals in environmental and wastewaters based on ultra high performance liquid chromatography (UHPLC)-tandem mass spectrometry (MS/MS). Chemosphere 85, 1390-1399, doi:10.1016/j.chemosphere.2011.07.071 (2011).
46. Low, K. et al. Prevalence and risk assessment of antibiotics in riverine estuarine waters of Larut and Sangga Besar River, Perak. Journal of Oceanology and Limnology 39, 122-134, doi:10.1007/s00343-020-9246-y (2021).
47. Luo, Y. et al. Occurrence and transport of tetracycline, sulfonamide, quinolone, and macrolide antibiotics in the Haihe River Basin, China. Environ Sci Technol 45, 1827-1833, doi:10.1021/es104009s (2011).
48. Managaki, S., Murata, A., Takada, H., Tuyen, B. C. & Chiem, N. H. Distribution of Macrolides, Sulfonamides, and Trimethoprim in Tropical Waters: Ubiquitous Occurrence of Veterinary Antibiotics in the Mekong Delta. Environmental Science & Technology 41, 8004-8010, doi:10.1021/es0709021 (2007).
49. Osorio, V., Pérez, S., Ginebreda, A. & Barceló, D. Pharmaceuticals on a sewage impacted section of a Mediterranean River (Llobregat River, NE Spain) and their relationship with hydrological conditions. Environ Sci Pollut Res Int 19, 1013-1025, doi:10.1007/s11356-011-0603-4 (2012).

50. Paíga, P. et al. Presence of pharmaceuticals in the Lis river (Portugal): Sources, fate and seasonal variation. Sci Total Environ 573, 164-177, doi:10.1016/j.scitotenv.2016.08.089 (2016).
51. Rivera-Jaimes, J. A. et al. Study of pharmaceuticals in surface and wastewater from Cuernavaca, Morelos, Mexico: Occurrence and environmental risk assessment. Science of The Total Environment 613-614, 1263-1274, doi:10.1016/j.scitotenv.2017.09.134 (2018).
52. Sharma, B. M. et al. Health and ecological risk assessment of emerging contaminants (pharmaceuticals, personal care products, and artificial sweeteners) in surface and groundwater (drinking water) in the Ganges River Basin, India. Science of The Total Environment 646, 1459-1467, doi:10.1016/j.scitotenv.2018.07.235 (2019).
53. Shimizu, A. et al. Ubiquitous occurrence of sulfonamides in tropical Asian waters. Science of The Total Environment 452-453, 108-115, doi:10.1016/j.scitotenv.2013.02.027 (2013).
54. Sim, W.-J., Lee, J.-W. & Oh, J.-E. Occurrence and fate of pharmaceuticals in wastewater treatment plants and rivers in Korea. Environmental Pollution 158, 1938-1947, doi:10.1016/j.envpol.2009.10.036 (2010).
55. Sörengård, M. et al. Mass loads, source apportionment, and risk estimation of organic micropollutants from hospital and municipal wastewater in recipient catchments. Chemosphere 234, 931-941, doi:10.1016/j.chemosphere.2019.06.041 (2019).
56. Spongberg, A. L. et al. Reconnaissance of selected PPCP compounds in Costa Rican surface waters. Water Research 45, 6709-6717, doi:10.1016/j.watres.2011.10.004 (2011).
57. Stipaničev, D., Dragun, Z., Repec, S., Rebok, K. & Jordanova, M. Broad spectrum screening of 463 organic contaminants in rivers in Macedonia. Ecotoxicology and Environmental Safety 135, 48-59, doi:10.1016/j.ecoenv.2016.09.004 (2017).
58. Tamtam, F. et al. Occurrence and fate of antibiotics in the Seine River in various hydrological conditions. Sci Total Environ 393, 84-95, doi:10.1016/j.scitotenv.2007.12.009 (2008).
59. ter Laak, T. L., van der Aa, M., Houtman, C. J., Stoks, P. G. & van Wezel, A. P. Relating environmental concentrations of pharmaceuticals to consumption: A mass balance approach for the river Rhine. Environment International 36, 403-409, doi:10.1016/j.envint.2010.02.009 (2010).
60. Thomas, K. V. & Hilton, M. J. The occurrence of selected human pharmaceutical compounds in UK estuaries. Marine Pollution Bulletin 49, 436-444, doi:10.1016/j.marpolbul.2004.02.028 (2004).
61. Tong, C., Zhuo, X. & Guo, Y. Occurrence and Risk Assessment of Four Typical Fluoroquinolone Antibiotics in Raw and Treated Sewage and in Receiving Waters in Hangzhou, China. Journal of Agricultural and Food Chemistry 59, 7303-7309, doi:10.1021/jf2013937 (2011).
62. Valdés, M. E., Amé, M. V., Bistoni Mde, L. & Wunderlin, D. A. Occurrence and bioaccumulation of pharmaceuticals in a fish species inhabiting the Suquía River basin (Córdoba, Argentina). Sci Total Environ 472, 389-396, doi:10.1016/j.scitotenv.2013.10.124 (2014).
63. Vieno, N. M., Tuhkanen, T. & Kronberg, L. Analysis of neutral and basic pharmaceuticals in sewage treatment plants and in recipient rivers using solid phase extraction and liquid chromatography–tandem mass spectrometry detection. Journal of Chromatography A 1134, 101-111, doi:10.1016/j.chroma.2006.08.077 (2006).
64. Vilimanovic, D., Andaluri, G., Hannah, R., Suri, R. & MacGillivray, A. R. Occurrence and aquatic toxicity of contaminants of emerging concern (CECs) in tributaries of an urbanized section of the Delaware River Watershed. AIMS Environmental Science 7, 302-319, doi:10.3934/environsci.2020019 (2020).
65. Wagil, M. et al. Development of sensitive and reliable LC-MS/MS methods for the determination of three fluoroquinolones in water and fish tissue samples and preliminary environmental risk assessment of their presence in two rivers in northern Poland. Sci Total Environ 493, 1006-1013, doi:10.1016/j.scitotenv.2014.06.082 (2014).

66. Wille, K. et al. Validation and application of an LC-MS/MS method for the simultaneous quantification of 13 pharmaceuticals in seawater. Anal Bioanal Chem 397, 1797-1808, doi:10.1007/s00216-010-3702-z (2010).
67. Zhang, L. et al. Occurrence, distribution, and ecological risk of pharmaceuticals in a seasonally ice-sealed river: From ice formation to melting. Journal of Hazardous Materials 389, 122083, doi:10.1016/j.jhazmat.2020.122083 (2020).
68. Oldenkamp, R. et al. A High-Resolution Spatial Model to Predict Exposure to Pharmaceuticals in European Surface Waters: ePiE. Environmental Science & Technology 52, 12494-12503, doi:10.1021/acs.est.8b03862 (2018).
69. Johnson, A. C., Ternes, T., Williams, R. J. & Sumpter, J. P. Assessing the Concentrations of Polar Organic Microcontaminants from Point Sources in the Aquatic Environment: Measure or Model? Environmental Science & Technology 42, 5390-5399, doi:10.1021/es703091r (2008).
70. Grill, G. et al. Estimating the eco-toxicological risk of estrogens in China's rivers using a high-resolution contaminant fate model. Water Research 145, 707-720, doi:10.1016/j.watres.2018.08.053 (2018).
71. Ehalt Macedo, H., Lehner, B., Nicell, J. & Grill, G. HydroFATE (v1): a high-resolution contaminant fate model for the global river system. Geosci. Model Dev. 17, 2877-2899, doi:10.5194/gmd-17-2877-2024 (2024).
72. Khan, U. Pharmaceutically Active Compounds in the Environment: Risks, Trade-offs and Sewer Epidemiology Doctor of Philosophy thesis, McGill University, <https://escholarship.mcgill.ca/concern/theses/m900nx65x> (2015).
73. Straub, J. O. Aquatic environmental risk assessment for human use of the old antibiotic sulfamethoxazole in Europe. Environ Toxicol Chem 35, 767-779, doi:10.1002/etc.2945 (2016).
74. Font, C., Bregoli, F., Acuña, V., Sabater, S. & Marcé, R. GLOBAL-FATE (version 1.0.0): A geographical information system (GIS)-based model for assessing contaminants fate in the global river network. Geosci. Model Dev. 12, 5213-5228, doi:10.5194/gmd-12-5213-2019 (2019).
75. Oldenkamp, R., Beusen, A. H. W. & Huijbregts, M. A. J. Aquatic risks from human pharmaceuticals—modelling temporal trends of carbamazepine and ciprofloxacin at the global scale. Environmental Research Letters 14, 034003, doi:10.1088/1748-9326/ab0071 (2019).
76. Strokal, M. et al. Global multi-pollutant modelling of water quality: scientific challenges and future directions. Current Opinion in Environmental Sustainability 36, 116-125, doi:10.1016/j.cosust.2018.11.004 (2019).
